# The first description of complete invertebrate arginine metabolism pathways implies dose-dependent pathogen regulation in *Apostichopus japonicus*

**DOI:** 10.1038/srep23783

**Published:** 2016-04-01

**Authors:** Shao Yina, Li Chenghua, Zhang Weiwei, Wang Zhenhui, Lv Zhimeng

**Affiliations:** 1 School of Marine Sciences, Ningbo University, Ningbo, Zhejiang Province 315211, P. R. China.

## Abstract

In this study, three typical members representative of different arginine metabolic pathways were firstly identified from *Apostichopus japonicus*, including nitric oxide synthase (NOS), arginase, and agmatinase. Spatial expression analysis revealed that the *AjNOS* transcript presented negative expression patterns relative to those of *Ajarginase* or *Ajagmatinase* in most detected tissues. Furthermore, *Vibrio splendidus*-challenged coelomocytes and intestine, and LPS-exposed primary coelomocytes could significantly induce *AjNOS* expression, followed by obviously inhibited *Arginase* and *AjAgmatinase* transcripts at the most detected time points. Silencing the three members with two specific siRNAs *in vivo* and *in vitro* collectively indicated that *AjNOS* not only compete with *Ajarginase* but also with *Ajagmatinase* in arginine metabolism. Interestingly, *Ajarginase* and *Ajagmatinase* displayed cooperative expression profiles in arginine utilization. More importantly, live pathogens of *V. splendidus* and *Vibrio parahaemolyticus* co-incubated with primary cells also induced NO production and suppressed arginase activity in a time-dependent at an appropriate multiplicity of infection (MOI) of 10, without non-pathogen *Escherichia coli*. When increasing the pathogen dose (MOI = 100), arginase activity was significantly elevated, and NO production was depressed, with a larger magnitude in *V. splendidus* co-incubation. The present study expands our understanding of the connection between arginine’s metabolic and immune responses in non-model invertebrates.

L-arginine is a crucial amino acid because it is involved in multiple metabolic pathways^1^,^2^, and plays important roles in physiological and pathological processes by producing a wide range of metabolites, including nitric oxide (NO), urea, creatinine, agmatine and polyamines^3^,^4^. It is therefore no surprise that its metabolic pathways are complex and highly regulated by different metabolites. The emerging importance of arginine is evident in many metabolic processes, such as the NO and polyamine biosynthesis pathways, in which arginine acts as a pivotal immune system regulator and helps modulate the immune response during infection^5^,^6^,^7^. Among them, *nitric oxide synthase* (*NOS*) and *arginase* are the most important enzymes that participate in inimitable catalytic steps with antagonistic roles linking arginine metabolism and the immune response^8^,^9^.

*NOS* and *arginase*, two classic immune regulator molecules, both have L-arginine as a common substrate and compete with each other for this substrate. Their involvement in arginine metabolism has been well described in mammalian immune systems^7^,^10^,^11^,^12^,^13^. There are three distinct isoforms of *NOS*, namely endothelial *NOS* (*eNOS*), neuronal *NOS* (*nNOS*) and inducible *NOS* (*iNOS*)^7^,^10^. *eNOS* and *nNOS* are continuously expressed and regulated by Ca^2+^/Calmodulin^14^. The third type of *iNOS* is not continuously presented but is highly induced by pathogens or bacterial components, such as lipopolysaccharide (LPS) and immunostimulation, with Ca^2+^-independent regulation^15^,^16^. NO is the central component produced by three isoforms of the NOSs using L-arginine as the exclusive physiological substrate and L-citrulline as a co-product^10^,^11^. It is both a gasotransmitter and an important signaling molecule, which is predominantly associated with antimicrobials in the immune system and is biosynthesized in many immunocytes, including macrophages, neutrophils, monocytes, and endothelial cells^17^,^18^,^19^,^20^. The depletion of arginine as a means of increasing NO production is a beneficial strategy employed by host cells in order to kill invasive bacteria, viruses and parasites^17^,^21^,^22^. In recent years, the three isoforms of NOSs have been obtained and described from many vertebrate species^23^,^24^, whereas only one *NOS* gene has been reported in most invertebrate genomes^21^,^25^. In marine invertebrates, the *NOS* gene has been identified from shrimp *Litopenaeus vannamei* and scallop *Chlamys farreri* after LPS or *Vibrio harveyi* exposure, and their roles in immune defense are well indicated^26^,^27^,^28^. Unfortunately, no evidence shows the other pathways of arginine metabolism, such as the *arginase* pathway, and whether it competes with the *NOS*/NO pathway is still largely a mystery in invertebrates.

In vertebrates, if the arginine metabolic pathway is controlled by *arginase*, the results would be completely opposite. *Arginase*, which has two isoforms (*arginase* I and *arginase* II), is one of the enzymes that competes with *NOSs* for L-arginine, which is a substrate produces ornithine and urea and reciprocally modulates *NOS* activity^29^. The hydrolysis of arginine through the *arginase* pathway will result in polyamine biosynthesis and lead to decreased bactericidal NO production. Additionally, it will increase the growth of bacterial and parasitic pathogens because polyamines play an important role in cell growth and proliferation, which is harmful to host tissues and cells^7^,^30^,^31^. Bussiere *et al*. demonstrated that the metabolite of spermine from the *arginase* pathway could prevent the antimicrobial effects of NO by inhibiting *iNOS* translation in macrophages infected by *Helicobacter pylori*^32^. Amazingly, *H. pylori* could use its own *arginase* (*RocF*) to evade the antimicrobial effects of macrophage-derived NO through competition using the common substrate L-arginine from the host^33^. Numerous studies have indicated that *arginase* and *NOS* play antagonistic roles during the immune response^8^,^31^. Inhibition or knockout of *arginase* is known to significantly increase NO production, which depends on *NOS*^3^,^34^. Interestingly, the metabolism of L-arginine to polyamines via *agmatinase* is an alternative pathway long recognized in lower organisms, which first degrades arginine to agmatine via *arginine decarboxylase* (*ADC*)^35^,^36^. Agmatinase is a binuclear manganese metalloenzyme and belongs to the ureohydrolase superfamily, which includes arginase, formiminoglutamase and proclavaminate amidino hydrolase^37^. It is well known that *agmatinase* shares regions of strong sequence homology with authentic *arginase* and acts as an intermediate in arginine metabolism of various lower organisms and mammals^38^,^39^. Agmatine not only competitively inhibits three isoenzymes of NOSs but also significantly inhibits polyamine synthesis catalyzed by *agmatinase*^40^,^41^,^42^. Currently, the metabolic responses of arginine and the modulation of its production through the *NOS/*NO or *arginase/*polyamines pathways, as well as the alternative pathway, have been well documented in vertebrate immune response, especially in mice and humans. However, whether these differentially expressed molecules exist in invertebrates remains largely unknown, and their roles acting in arginine metabolism under pathogen infection or disease outbreaks are poorly understood. Thus, the exploration of complete arginine metabolic pathways in invertebrates is significant.

The invertebrate sea cucumber *Apostichopus japonicus* (Echinodermata, Holothuroidea), which has an innate immune system, is one of the most important economic marine species in Chinese aquaculture. In echinoderms, cell-based immunity is based on coelomocytes, a morphologically heterogeneous population with the capacity to recognize and neutralize pathogens. Unfortunately, the natural resources of *A. japonicus* in China have declined drastically due to various viral and bacterial disease outbreaks^43^,^44^ in which *Vibrio* was widely accepted as one of the major pathogens by many researchers, especially *Vibrio splendidus* and *Vibrio parahaemolyticus*. They are both Gram-negative halophilic and mesophilic bacteria and are commonly found in marine environments, causing diseases in marine animals^45^. In our previous work, we found that the metabolites of arginine uniquely increased in *V. splendidus*-challenged *A. japonicus* samples after infection for 96 h, whereas lower levels were detected in SUS-diseased sea cucumbers^46^. It is important to not only investigate the mechanisms of initiating an immune response but also gain a deeper understanding of the reasons why these reactions appear. Therefore, in our current study, we will first describe the three arginine metabolic pathways in sea cucumbers and understand their functional cooperation in allocating arginine during pathogen infection.

## Results

### Cloning and sequences analysis of the three genes

Three full-length cDNAs from the different arginine pathways were generated by overlapping the fragments from ESTs and using the RACE approach in the sea cucumber *Apostichopus japonicus* (denoted *AjNOS*, *Ajarginase*, and *Ajagmatinase*), which were deposited in GenBank with accession Nos. KT366016, KT724965 and KT366017, respectively. The cDNA sequence of *AjNOS* was 5957 bp in length and contained an ORF of 5313 bp encoding a predicted product with 1770 amino acid residues with a molecular weight of 197.02 kDa and a theoretical *pI* of 6.23 (Fig. S1). The 5′-UTR was 68 bp long, and the 3′-UTR of 576 bp contained a polyadenylation signal (AATAAA) and RNA instability sequences (ATTTA). Multiple alignments showed that the deduced amino acid sequence of AjNOS shared greater identity with the NOS family, such as 52% homology with *Strongylocentrotus purpuratus* NOS (XP_011665134.1), 52% homology with *Homo sapiens* eNOS (AAH69465.1), 46% homology with *H. sapiens* iNOS (BAA37123.1), and 45% homology with *H. sapiens* nNOS (AAB60654.1). The representative domains of NOS from low invertebrate to human were totally conserved in the deduced amino acid of AjNOS, including the PDZ domain, N-terminal oxygenase domain, C-terminal reductase domain and a CaM binding site (Fig. S2). The *Ajarginase* cDNA transcript was 1860 bp and consisted of an ORF encoding a protein sequence of 384 amino acid residues with a predicted molecular weight of 41.71 kDa, and a theoretical *pI* of 6.54 (Fig. S3). The *AjAagmatinase* cDNA transcript comprised 1411 bp, including a 246 bp 5′-UTR, a 103 bp 3′-UTR with one RNA instability sequence (ATTTA) encoding 353 amino acid residues (Fig. S4). The predicted molecular mass of the deduced amino acids of Ajagmatinase was 38.64 kDa, and its theoretical *pI* was 6.46. Amino acid sequences alignment between Ajarginase and Ajagmatinase both from the ureohydrolase superfamily, showed only 18% identity with the highly conserved region of an arginase domain (Fig. S5). Among the conserved region are the two manganese-ion-binding sites present in these proteins. Sea cucumber agmatinase had the highest amino acid identity and similarity with *C. gigas* agmatinase (XP_011443425.1), although the overall sequence homology with human arginase 1 and arginase 2 was only 21% and 18%, respectively. To determine the evolutionary position of AjNOS, Ajarginase and Ajagmatinase from different arginine metabolic pathways, phylogenetic trees were constructed using the NJ method. The results showed that all three typical members shared greater homology to their counterparts from vertebrates. AjNOS was first clustered with other invertebrates from *S. purpuratus*, *C. gigas* (XP_011420158.1) and *Aplysia california* (NP_001191470.1), and then clustered with vertebrate nNOS to form a separated clade, which indicated that AjNOS belong to the nNOS subfamily (Fig. S6). Ajagmatinase was first grouped with invertebrate and vertebrate agmatinase and formed an independent clade; it was then grouped with bacteria agmatinase, which were clearly distinguished from the arginase subfamily, with the *Vibrio parahaemolyticus* arginase used an out group (Fig. S7). This phylogenetic tree analysis suggested that arginases and agmatinases from different species were derived from a common ancestor.

### Tissue distribution of *AjNOS*, *Ajarginase* and *Ajagmatinase*

**Figure 1:**
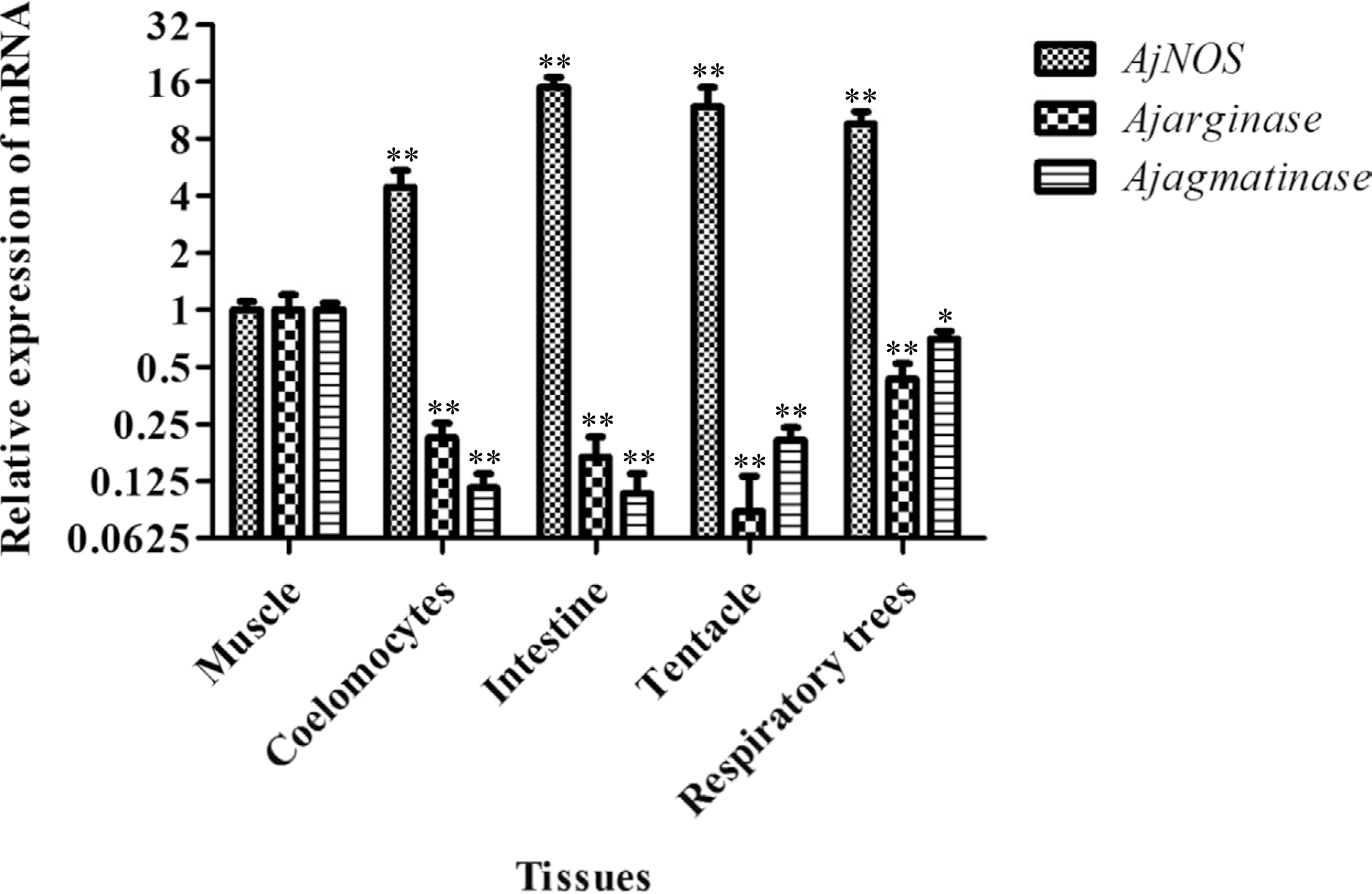
The tissue distribution of *AjNOS*, *Ajarginase* and *Ajagmatinase* in normal sea cucumber detected by quantitative PCR. The transcript levels in coelomocytes, intestine, tentacle, and respiratory tress were normalized to that in muscle. Five biological replicates were performed in the experiment and the obtained data were expressed as the mean ± SD (n = 5). Asterisks indicated significant differences: *P < 0.05, **P < 0.01.

The constitutive expression of *AjNOS*, *Ajarginase* and *Ajagmatinase* in different tissues was investigated by quantitative real-time PCR, and the expression levels in muscle served as a reference. The results showed that the three genes were ubiquitously expressed in all examined tissues (Fig. 1). The mRNA transcript of *AjNOS* was expressed most strongly in the intestine (14.95-fold), followed by the tentacle (11.82-fold), respiratory tree (9.58-fold) and coelomocytes (4.43-fold). *Ajarginase* and *Ajagmatinase* displayed almost opposite expression profiles as *AjNOS*. *Ajarginase* and *Ajagmatinase* both presented relative lower expression levels in four other tissues including coelomocytes (0.21-fold and 0.12-fold), intestine (0.17-fold and 0.11-fold), tentacle (009-fold and 0.20-fold), and respiratory tree (0.43-fold and 0.74-fold) when compared with muscle.

### Time-course expression of *AjNOS*, *Ajarginase* and *Ajagmatinase in vivo* and *in vitro*

**Figure 2:**
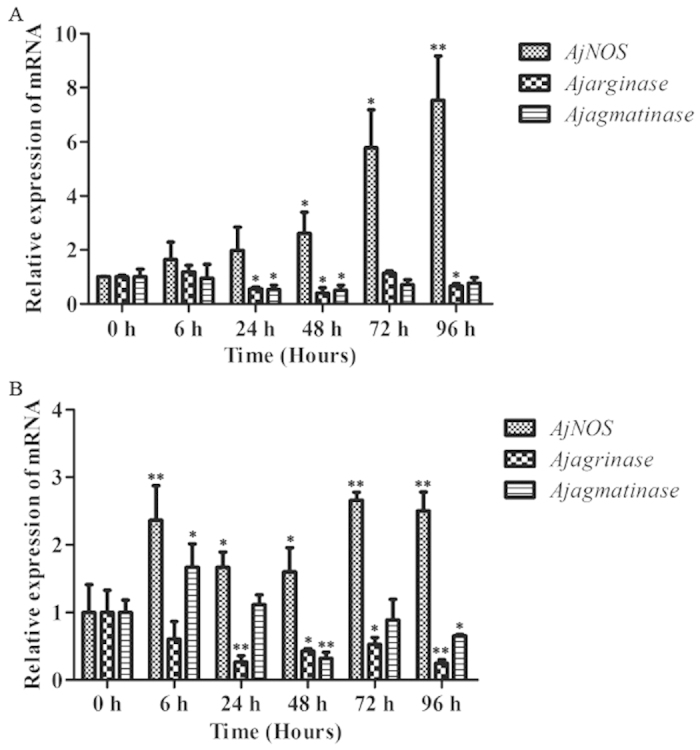
Time-course expression of *AjNOS*, *Ajarginase* and *Ajagmatinase* in coelomocytes and intestine after *Vibrio splendidus* infection. Five biological replicates were performed in the experiment and the obtained data were expressed as the mean ± SD (n = 5). Asterisks indicated significant differences: *P < 0.05, **P < 0.01.

After the stimulation of *V. splendidus*, the temporal mRNA expression of *AjNOS*, *Ajarginase* and *Ajagmatinase* in coelomocytes and intestine is shown in Fig. 2. The expression level of *AjNOS* mRNA in coelomocytes (Fig. 2A) was gradually increased at 24 h post-infection, which sharply increased and reached peak expression at 96 h with a 7.53-fold (*P* < 0.01) increase compared to that in the control group throughout the experiment. The expression profiles of *Ajarginase* and *Ajagmatinase* were completely different from that of *AjNOS*. The mRNA expression levels of *Ajarginase* and *Ajagmatinase* remained at the control level at the first 6 h. Subsequently, the mRNA levels of *Ajarginase* and *Ajagmatinase* were both fleetly decreased and reached their lowest mRNA levels at 48 h with 0.40-fold (*P* < 0.05) and 0.50-fold (*P* < 0.05) decreases, respectively, which were still lower than the original level, with a lower magnitude in *Ajarginase* until the end of the test compared with the control group. When in the intestine (Fig. 2B), the level of the *AjNOS* transcript was also significantly increased at each detected time point compared with controls and occurred earlier than that of coelomocytes after *V. splendidus* infection, although the increased magnitude was lower than in coelomocytes. Consistently, the expression level of *Ajarginase* was tightly correlated with that of the coelomocyte *Ajarginase*, which also presented an opposite trends of expression to that of *AjNOS* in intestinal tissue. In contrast, *Ajagmatinase* was expressed at a higher level at 6 h, with a 1.67-fold (*P* < 0.05) compared with the control. Moreover, at 48 h, its expression was decreased 0.32-fold (*P* < 0.05), but it was still lower than the original level at 96 h compared with the control group.

**Figure 3:**
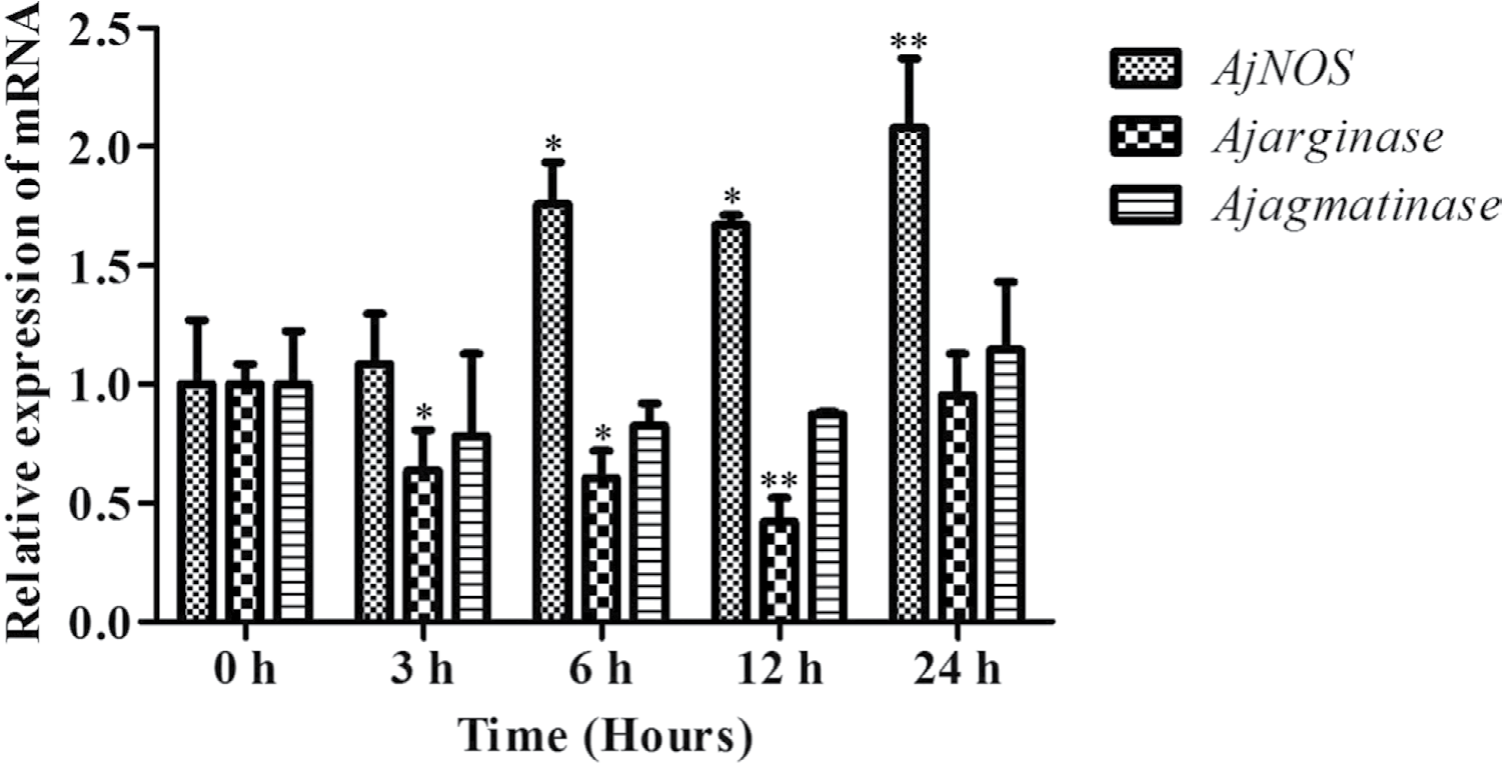
Transcriptional regulation of *AjNOS*, *Ajarginase* and *Ajagmatinase* in LPS-exposed coelomocytes at 0, 3, 6, 12 and 24 h. Five biological replicates were performed in the experiment and the obtained data were expressed as the mean ± SD (n = 5). Asterisks indicated significant differences: *P < 0.05, **P < 0.01.

The expression profiles of the three genes in primary coelomocytes after exposure to LPS *in vitro* were summarized in Fig. 3. At an LPS concentration of 10 μg mL^−1^, transcription of *AjNOS* remained at the control level for the first 3 h, then increased significantly (1.76-fold, *P* < 0.05) at 6 h and reached the highest expression at 24 h (2.08-fold, *P* < 0.01) compared with the control group. The level of the *Ajarginase* transcript was down regulated after 3 h (0.64-fold, *P* < 0.05) and reached its lowest expression at 12 h with a 0.42-fold decrease (*P* < 0.01). However, the expression profile of *AjAgmatinase* was not significantly changed at all examined time points during LPS challenge.

### NO production and arginase activities following *AjNOS*, *Ajarginas*e or *Ajagmatinase* silencing *in vitro*

**Figure 4:**
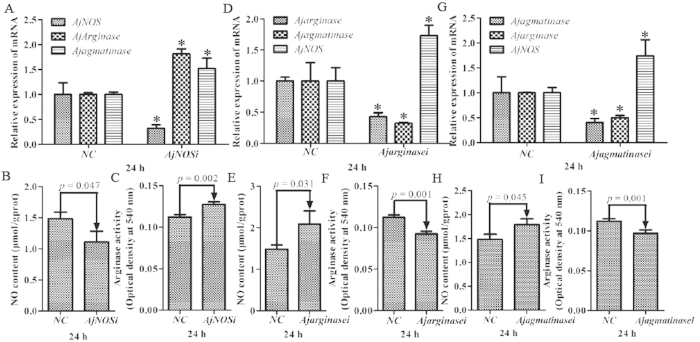
Related data from *Apostichopus japonicus* primary cultured coelomocytes after each gene silencing. (**A**,**D**,**G**) Silencing efficiency of *AjNOS*, *Ajarginase* or *Ajagmatinase* in primary coelomocytes after specific siRNAs transfection and relative expression of mRNAs after interfering for 24 h, respectively. (**B**,**E**,**H**) NO production in the primary cultured coelomocytes after *AjNOS*, *Ajarginase* or *Ajagmatinase* knock-down, respectively. (**C**,**F**,**I**) arginase activity in the primary cultured coelomocytes after *AjNOS*, *Ajarginase* or *Ajagmatinase* knock-down, respectively. Five biological replicates were performed in the experiment and the obtained data were expressed as the mean ± SD (n = 5).

We transfected two specific siRNAs for each gene, and the efficiency of RNAi-mediated transcript depletion in primary cells was determined by quantitative RT-PCR. The interference efficiency of each gene, NO production and arginase activity were taken as the average after two siRNA transfections. Our results showed that *AjNOS*, *Ajarginase* and *AjAgmatinase* mRNA transcripts were inhibited by more than 52% after specific siRNA transfection (Fig. 4A,D,G). For *AjNOS* interference, the NO content of primary cells was markedly decreased by 25.1% (*P *=* *0.047) compared with the negative control (Fig. 4B). In contrast, the mRNA expression of *Ajarginase* and *Ajagmatinase* was significantly increased by 1.81 fold (*P *<* *0.05) and 1.52-fold (*P *<* *0.05) (Fig. 4A), respectively, and the arginase activity also showed a positive correlation with the mRNA expression, which increased by 13.4% (*P *=* *0.002) after *AjNOS* knock-down (Fig. 4C). Moreover, the *AjNOS* transcript was dramatically up-regulated 1.73-fold (*P *<* *0.05) and 1.74-fold (*P *<* *0.05), and the NO production increased by 40.6% (*P *=* *0.031) and 20.6% (*P *=* *0.045) after *Ajarginase* and *Ajagmatinase* silencing for 24 h, respectively (Fig. 4E,H). Arginase activity was tightly correlated with the expression levels of *Ajarginase* and *Ajagmatinase* and decreased by 17.7% (*P *=* *0.001) and 13.5% (*P *=* *0.001), respectively, after interference (Fig. 4F,I).

### NO production and arginase activities following *AjNOS*, *Ajarginase* or *Ajagmatinase* silencing *in vivo*

**Figure 5:**
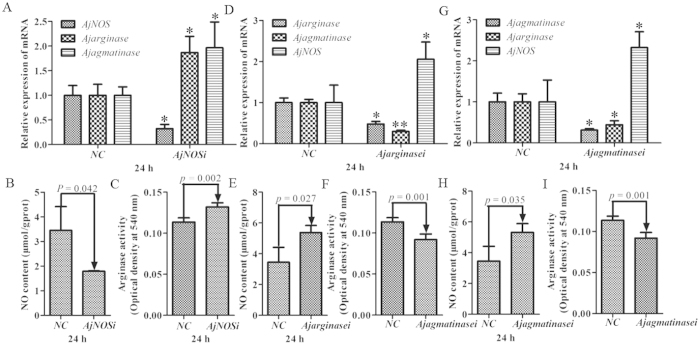
Related data from *Apostichopus japonicus* individual levels after each gene silencing. (**A**,**D**,**G**) Silencing efficiency of *AjNOS*, *Ajarginase* or *Ajagmatinase* in individuals coelomocytes after specific siRNAs transfection and relative expression of mRNAs after interfering for 24 h, respectively. (**B**,**E**,**H**) NO production in the individuals coelomocytes after *AjNOS*, *Ajarginase* or *Ajagmatinase* knock-down, respectively. (**C**,**F**,**I**) arginase activity in the individuals coelomocytes after *AjNOS*, *Ajarginase* or *Ajagmatinase* knock-down, respectively. Five biological replicates were performed in the experiment and the obtained data were expressed as the mean ± SD (n = 5).

Figure 5 shows the mRNA expression levels of *AjNOS*, *Ajarginase* and *Ajagmatinase*, as well as NO production and arginase activity after the silencing of three genes in individual coelomocytes. The data were processed *in vivo* as well as *in vitro*. Three genes were significantly inhibited by more than 55% after specific siRNA transfection (Fig. 5A,D,G). The results in Fig. 5A showed that the levels of *Ajarginase* and *Ajagmatinase* were increased by 1.87-fold (*P *<* *0.05) and 1.97-fold (*P *<* *0.05), respectively, in the *AjNOS* silenced sea cucumbers, followed by the decrease in NO generation by 48.0% (*P *=* *0.042) and an increase in arginase activity by 16.1% (*P *=* *0.002) (Fig. 5B,C). When the sea cucumbers were treated with *Ajarginase* or *Ajagmatinase* siRNAs, the experimental results showed that the level of *AjNOS* was markedly increased by 2.06-fold (*P *<* *0.05) and 2.32-fold (*P *<* *0.05) after *Ajarginase* or *Ajagmatinase* siRNAs, respectively, were injected into individuals (Fig. 5D,G). Meanwhile, the amount of NO production was increased by 55.9% (*P *=* *0.027) and 49.9% (*P *=* *0.035) in silenced sea cucumbers (Fig. 5E,H), and arginase activity was observed to be lower both in the *Ajarginase* (decreased by 23.6%, *P *=* *0.001) and *Ajagmatinase* (decreased by 19.1%, *P *=* *0.001) siRNA groups after 24 h silencing compared with the negative control (Fig. 5F,I). Moreover, the *Ajarginase* and *Ajagmatinase* transcripts displayed cooperative expression profiles after one of interference both *in vivo* and *in vitro*, respectively.

### *In vitro* induction of NO production and arginase activity following pathogenic or non-pathogenic challenge

**Figure 6:**
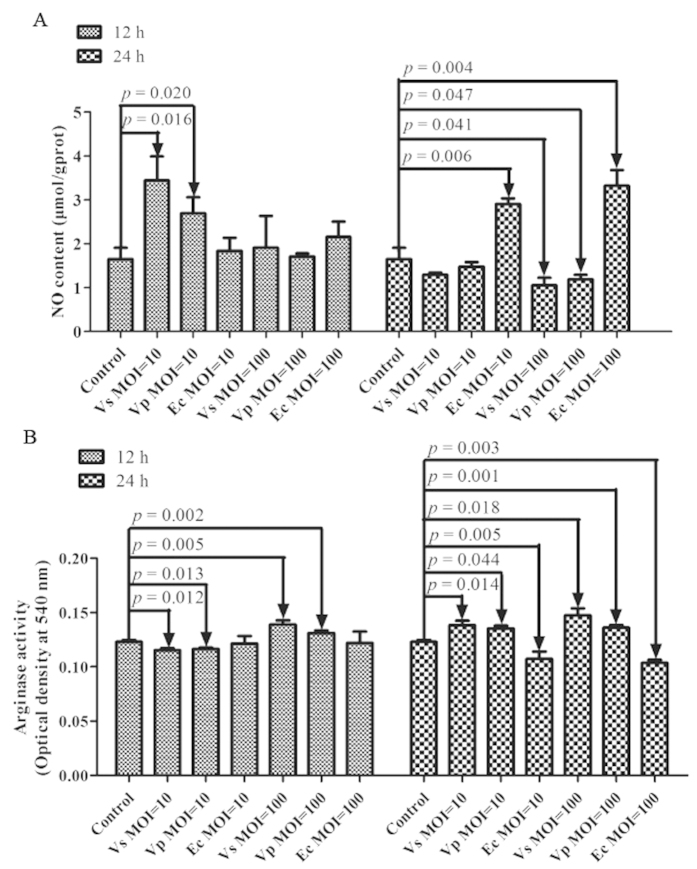
Effects of *in vitro* stimulation of pathogen or non-pathogen on *Apostichopus japonicus* primary cultured coelomocytes for 12 and 24 h with an appropriate multiplicity of infection (MOI) of 10 and 100. (**A**) NO production in the primary cultured coelomocytes after *Vibrio splendidus*, *Vibrio Parahaemolyticus* and *Escherichia Coli* infection. (**B**) arginase activity in the primary cultured coelomocytes after *Vibrio splendidus*, *Vibrio Parahaemolyticus* and *Escherichia Coli* infection. Five biological replicates were performed in the experiment and the obtained data were expressed as the mean ± SD (n = 5).

The NO production and arginase activity upon primary coelomocytes stimulation with pathogens or non-pathogens were generally dependent on the MOI employed and time point (Fig. 6). When primary cells were co-incubated with the pathogens *V. splendidus* and *V. parahaemolyticus*, NO production was markedly increased by 108.8% (*P *=* *0.016) and 63.3% (*P *=* *0.020), respectively, at 12 h and recovered to the original level at 24 h with an MOI of 10 (Fig. 6A). When challenge with two pathogens at an MOI of 100, NO production was gradually decreased by 36.2% (*P *=* *0.041) and 27.8% (*P *=* *0.047) for 24 h, respectively. There was no significant change in NO production and arginase activity after non-pathogen challenge of primary cells with *E. coli* with different MOIs at 12 h. However, NO production was sharply increased by 76.3% (*P *=* *0.006) and 101.5% (*P *=* *0.004) after 24 h with MOI = 10 and MOI = 100, followed by down-regulated arginase activity with 12.7% (*P *=* *0.005)and 15.7% (*P *=* *0.003), respectively. Furthermore, arginase activity was also found to be negatively correlated with NO production after pathogen challenge of primary cells *in vitro* (Fig. 6B). The arginase activity of sea cucumber coelomocytes exhibited the highest degree of sensitivity to *V. splendidus*. At an MOI of 10, the arginase activity first decreased by 6.2% (*P *=* *0.012) in the *V. splendidus* group and 5.4% (*P *=* *0.013) in the *V. parahaemolyticus* group at 12 h, and both induced significant response rates in 12.8% (*P *=* *0.005) and 10.1% (*P *=* *0.002), respectively, at 24 h compared to controls. When an MOI of 100, arginase activity was quickly up-regulated by 12.5% (*P *=* *0.014) in the *V. splendidus* group and 6.7% (*P *=* *0.044) in the *V. parahaemolyticus* group at 12 h and increased by 19.8% (*P *=* *0.018) and 10.8% (*P *=* *0.001) at 24 h after *V. splendidus* and *V. parahaemolyticus*, respectively, was co**-incubated with primary cultured cells.

## Discussion

As a common substrate for a number of NO, urea, agmatine and polyamine biosynthetic pathways, arginine metabolism plays an irreplaceable role in cardiovascular function, neurotransmission, cell proliferation and immunity^4^,^6^,^47^. Today, the interaction between metabolism and the immune response is regulated by various arginine metabolic enzymes and constitutes an extremely intriguing area in vertebrates research. However, scarce information is available concerning the modulation of arginine metabolic pathways involved in the immune defense of invertebrates, particularly in non-model invertebrates. In this paper, we first focused on the metabolite of arginine with its key downstream molecules involved in different arginine metabolic pathways of sea cucumbers and their competitive roles in responding to the immune defense, in order to understand the crosstalk between the immune response and arginine metabolism in invertebrates.

Three typical members of *AjNOS*, *Ajarginase* and *Ajagmatinase* from representative arginine metabolic pathways were first isolated and characterized from the sea cucumber *A. japonicus*. Our results showed that the three genes were ubiquitously expressed in all examined tissues (Fig. 1), suggesting that they might be involved in versatile physiological processes^28^,^39^,^48^. The mRNA level of *AjNOS* was highly expressed in coelomocytes (4.43-fold), the respiratory tree (9.58-fold) and tentacles (11.82-fold) and was expressed most strongly in the intestine (14.95-fold). The tentacle, an important site of entry for microorganisms, especially Gram-negative pathogenic bacteria^49^, exhibited greater expression of *AjNOS*, suggesting that it plays important roles in defending against invading bacteria. Notably, the expression levels of *Ajarginase* and *Ajagmatinase* displayed almost completely opposite expression profiles compared with the *AjNOS* transcript, which was expressed at lower levels in other four tissues. These tissues expression profiles revealed that *AjNOS* might compete with *Ajarginase*, *Ajagmatinase* or both in the arginine metabolic pathways of sea cucumbers.

In most studies, haemocytes and the intestine are the most important immune tissues that play key roles in immune responses and have been used to investigate the fluctuation of immune-related genes^50^,^51^. Therefore, we were interested in the expression pattern of *AjNOS*, *Ajarginase* and *Ajagmatinase* mRNAs in *A. japonicus* coelomocytes and the intestine in response to bacterial infection *in vivo* and LPS challenge *in vitro*. The *AjNOS* transcript of coelomocytes was expressed significantly at 24 h and the expression level peaked at 96 h after *V. splendidus* stimulation compared with the control (Fig. 2). At the same time, we found that the mRNA expressions of *Ajarginase* and *Ajagmatinase* were both significantly inhibited at most detected time points of *AjNOS* induction. Similar results were also found in intestinal tissue, although the expression of *Ajagmatinase* was up-regulated 1.67-fold (*P* < 0.05) at 6 h after *V. splendidus* infection and its expression gradually decreased at the later time points. Consistently, the mRNA level of *AjNOS* in LPS challenge primary cells was also gradually up-regulated and reached its highest level at 24 h until the end of experiment. The *Ajarginase* transcript still showed opposite trends of expression to those of *AjNOS*, although the *Ajagmatinase* transcript was not significantly changed. The expression of the three genes in coelomocytes and the intestine changed rapidly and dynamically in response to the injection of *V. splendidus* or LPS, suggesting that they play important roles in sea cucumbers′ defense against pathogen infection. In vertebrates, *iNOS* could be induced by a variety of immune cells to synthesize high amounts of NO under immune defense^10^,^17^. We speculated that only one NOS isoform in *A. japonicus* exists, and it might possess multiple functions as a primordial NOS^52^. As an important cell signaling molecule, NO is often a major cytoprotective agent and controls the fate of pathogens in the immune system^22^,^53^,^54^. The generation of NO from arginine by *NOSs* is necessary for host cells to kill invading pathogens. On the contrary, the conversion of arginine to ornithine and urea in order to promote polyamine biosynthesis through the *arginase* pathway or alternative pathways via *agmatinase* can lead to the disruption of NO production of the host and enhance the survival of the bacteria^40^,^55^,^56^. Moreover, *N*^G^-hydroxy-L-arginine (NOHA), an intermediate in *NOS*, can accumulate sufficiently in *iNOS*-expressing cells, and it is a potent natural inhibitor of both *arginase* and arginase activity^57^,^58^. Based on a previous study, we surmised that the hydrolysis of increased arginine in different tissues of the sea cucumber was controlled by *AjNOS* under acute stress. Switching the immune response to the *AjNOS* reaction was required to reduce the bacteria infection.

To directly test the involvement of the arginine metabolic pathways in the sea cucumber, we used RNA interference technology to further confirm their competitive roles in arginine metabolism. siRNA-mediating RNAi is a common method of functional validation in many invertebrates *in vitro*, which has proven to be feasible and effective due to complex interplay of genetic and environmental influences in individual animals. In cultured cells, however, it is sometimes not stable *in vitro* and it does not approach stability to *in vivo*. Therefore, three molecules were blocked by transfecting specific siRNAs *in vivo* and *in vitro*, respectively. Our results indicated that the inhibition of *Ajarginase* or *Ajagmatinase in vivo* and *in vitro*, both resulted in increased NO production by induced *AjNOS* (Figs 4 and 5). Whereas, the expression profiles of *Ajarginase* and *Ajagmatinase* were up-regulated after *AjNOS* knock-down both *in vivo* and *in vitro*, accompanied by suppressed NO generation. Wijnands^13^ study demonstrated that *arginase*-I deficiency in murine endothelial cells and macrophages resulted in increased NO production by *NOS* during endotoxemia. In our case, the enhanced NO production in *Ajarginase* or *Ajagmatinase* inhibited by specific siRNAs was largely derived from *AjNOS*. Moreover, the arginase activity, a key parameter in the polyamine biosynthesis pathway and necessary for pathogen survival^59^,^60^, was significantly decreased after *Ajarginase* or *Ajagmatinase* knock-down and sharply increased after *AjNOS* silencing both *in vivo* and *in vitro*. There is strong evidence that constitutive levels of arginase activity generated from *arginase* in endothelial cells limit *NOS* and NO production^61^,^62^, which was in accordance with our results after *AjNOS* silencing. *Arginase* and *agmatinase* release urea and ornithine and putrescine, respectively, which differ from each other only by the presence of substrates. It is clear that *agmatinase* catalyze the hydrolysis of agmatine to urea and putrescine, participating in an alternative pathway for polyamine biosynthesis, while functioning as *arginase*^35^,^63^,^64^. Undoubtedly, *arginase* and *NOS* compete with each other in using arginine as a common substrate^65^,^66^. In our study, *Ajagmatinase* might also modulate arginase activity in the alternative pathway by competing with arginine and *AjNOS*. The findings shown here identify that *AjNOS* not only compete with *Ajarginase* but also with *Ajagmatinase* in arginine metabolism of the sea cucumber. This is first evidence that the alternative pathway modulated by *agmatinase* also plays an important role in arginine metabolism. In addition, we found that the *Ajagmatinase* transcript was suppressed after *Ajarginase* knock-down, and the *Ajarginase* mRNA level also dropped after *Ajagmatinase* interference both *in vivo* and *in vitro*. These results showed that both *Ajarginase* and *Ajagmatinase* need each other in arginine metabolism when competing with *AjNOS*.

To better understand the substrate competition among different arginine metabolic pathways in invertebrates responding to immune defense, we examined NO production and arginase activity from primary sea cucumber cells co-incubated with pathogens or non-pathogens at different doses and different time points. Our findings revealed that loss of arginase activity resulted in NO production, which was generally dependent on the MOI employed and time points of infection with *V. splendidus* and *V. parahaemolyticus*, especially for *V. splendidus* (Fig. 6). Although, primary cells challenged with non-pathogen *E. coli* showed NO production was significantly increased at 24 h with different MOIs followed by decreased arginase activity, which indicated that the increased NO was also able to kill non-pathogen in immune response. At an MOI of 10 after 12 h, NO production was significantly induced by *AjNOS* in order to kill the invading *V. splendidus or V. parahaemolyticus*. However, high concentrations of NO had a stronger cytotoxicity during the immune response; it could not only eliminate the intrusive pathogens but also harm to normal host cells^67^,^68^. Thus, the NO concentration was recovered to the original level at 24 h, which was essential for the maintenance of immune homeostasis of sea cucumber primary cells. At an MOI of 100, with the arginase activity increasing both with *V. splendidus or V. parahaemolyticus* infection, we speculated that metabolism through the arginase pathway and alternative pathway was more dominant than the *NOS* pathway, which might favor bacterial invasion. Most studies demonstrated that polyamines generated from the *arginase* or *Agmatinase* pathway inhibited NO production and supported bacterial growth^30^,^31^. However, host arginase activity not only promoted the spread of pathogens^69^, but more importantly, pathogen could also use its own *arginase* to compete with L-arginine from the host^70^. Therefore, at an MOI of 100, we speculated that the *V. splendidus* or *V. parahaemolyticus* that escaped the immune response might directly use host arginine, leading to polyamines biosynthesis pathway with increased arginase activity.

**Figure 7:**
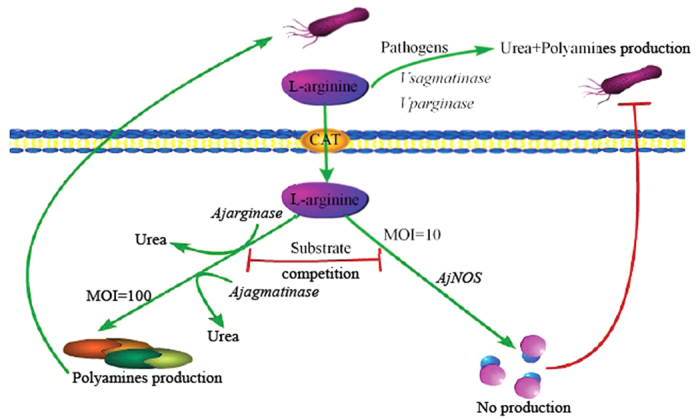
Schematic representation of the arginine metabolic pathways of host-pathogen interaction. CAT was short from cationic amino acid transporter and mediated arginine transport. The extracellular urea released by pathogens (*Vsagmatinase* or *Vparginase*). *Ajarginase* and *Ajagmatinase* transcribed and translated and generated into urea. *AjNOS* transcribed and translated and generated into NO.

In summary, the present study first depicted the different arginine metabolic pathways in sea cucumbers, and the arginine metabolic enzymes that modulate NO production and arginase activity in the immune response were schematically presented (Fig. 7). The cationic amino acid transporter (CAT) mediated L-arginine transport^4^. The most exciting findings in this field originated from our studies indicating that *AjNOS* not only competes with *Ajarginase* but also with *Ajagmatinase* in the arginine metabolism of sea cucumbers. Moreover, the substrate competition among *AjNOS*, *Ajarginase* and *Ajagmatinase* to allocate arginine convert in the generation of NO and arginase activity was generally dependent on the dose of bacteria and the time course of infection. In our case, the pathogens whether directly use host arginine to promote urea release and polyamine biosynthesis will be confirmed in next work.

## Methods

### Animals and challenge experiments

Sea cucumbers *A. japonicus* (weight 125 ± 15 g) were collected from Bowang Aquaculture Company (Ningbo, China) and reared in 30 L aerated natural seawater (salinity 28, temperature 16 °C) for 3 days. For the challenge experiments, sea cucumbers were randomly divided into four tanks, each containing twenty individuals. The three experimental groups were infected with live *V. splendidus* at a final concentration of 10^7^ CFU mL^−1^. The infection dose and sampling points were determined by immune genes expression analysis. Five individuals were randomly sampled at 6, 24, 48, 72 and 96 h post infection. The remaining twenty untreated sea cucumber served as control group and were collected at 0 h. Coelomic fluids were collected through a 300 Mesh CellCribble and then centrifuged at 800 × g for 5 min to harvest the coelomocytes along with the intestine for time-course expression analysis. For spatial expression analysis, coelomocytes, intestine tissue and three other tissues, including muscle, tentacle and respiratory trees, were collected from control individuals using sterilized scissors and tweezers. All tissues (*approx*. 100 mg wet weight) were ground into powder in liquid nitrogen using a mortar and a pestle. We performed 5 replicates in the experimental group as well as the control group and all samples were stored at −80 °C for further analysis.

### Rapid application of cDNA ends (RACE)

Partial sequences of *NOS*, *arginase* and *agmatinase* genes were generated by screening *A. japonicus* transcriptome database^71^. Gene-specific primers for three genes (Table 1) were designed based on the acquired unigenes and the full-length cDNA sequences were subsequently cloned using the 3′, 5′-Full RACE Kit (TaKaRa) following the manufacturer’s instructions. The desired PCR products were purified and then cloned into the pMD18-T simple vector (TaKaRa). Three positive clones for each product were sequenced at Sangon (Shanghai, China). The sequences were overlapped, verified, and subjected to cluster analysis.

### Sequence analysis

Sequences homology were obtained using BLAST program at National Centre for Biotechnology Information (http://www.ncbi.nlm.nih.gov/blast) and the deduced amino acid sequences of AjNOS, Ajarginase and Ajagmatinase were analyzed with the expert protein analysis system (http://www.expasy.org/). The molecular mass (MM) and theoretical *pI* of the protein were calculated based upon its deduced amino acids by the ProtParam tool (http://www.expasy.ch/tools/protparam.html). Domain in these amino acid sequences were detected using the simple modular architecture research tool (SMART) program (http://www.smart.emblheidelbergde/). Multiple alignments analysis of each protein were performed using the ClustalW2 Multiple Alignment program (http://www.ebi.ac.uk/clustalw/) and the Multiple Align Show program (http://www.bio-soft.net/sms/index.html). Phylogenetic and molecular evolutionary analyses were conducted using MEGA version 4.0 program.

### Quantitative real-time PCR analysis of *AjNOS*, *Ajarginase* and *Ajagmatinase* mRNAs expression

The tissue distribution and time-course expression of *AjNOS*, *Ajarginase* and *Ajagmatinase* were performed using a Rotor-Gene 6000 real-time PCR detection system. Total RNA was isolated from coelomocytes and other tissues using Trizol (Invitrogen), and cDNA was synthesized using the Primescript™ II 1st cDNA Synthesis Kit (Takara). The employed primers are listed in Table 1. Amplifications were carried out in a 20 μL reaction volume, containing 8 μL of the 1:100 diluted cDNA, 1 μL of each of the primers, and 10 μL of SYBR Green Mix (Takara). The reaction mixture was incubated for 5 min at 95 °C, followed by 40 amplification cycles of 15 s at 95 °C, 20 s at 60 °C, and 20 s at 72 °C. To maintain consistency, the baseline was set automatically by the software. The 2^−ΔΔCT^ method was used to analyze the relative expression level of the candidate genes^72^, and the value obtained denoted the n-fold difference relative to the calibrator. Quantitative data were expressed as the mean ± standard deviation (SD) of five biological replicates. One-way (ANOVA) was applied to discern significant differences between control and experimental groups. Any significant differences relative to the control for each time point were indicated with an asterisk at *P* < 0.05 and two asterisks at *P* < 0.01.

### Temporal expression profiles of *AjNOS*, *Ajarginase* and *Ajagmatinase* in LPS-exposed primary coelomocytes

Sea cucumbers (weight 125 ± 15 g) were dissected with sterilized scissors on ice as described^73^,^74^. In brief, the coelomic fluids were filtered through a 300 Mesh CellCribble to remove large tissue debris, mixed with the anticoagulant solution (0.02 M EGTA, 0.48 M NaCl, 0.019 M KCl, 0.068 M Tri-HCl, pH7.6) in a 1:1(V:V) ratio, and then centrifuged at 800 × g, 16 °C for 10 min. The harvested cells were washed twice with isotonic buffer (0.001 M EGTA, 0.53 M NaCl, 0.01 M Tris-HCl, pH7.6) and re-suspended in the Leiboviz’s L-15 cell culture medium (Invitrogen, USA) containing penicillin (100 U mL^−1^) and streptomycin sulfate (100 mg mL^−1^). The cells were diluted to 10^6^ cells mL^−1^ and transferred into a 24 well culture microplates and incubated in 16 °C for 12 h prior to lipopolysaccharides (Sigma, USA) exposure. For the LPS challenge, the cells were stimulated with 10 μg mL^−1^ LPS for 3, 6, 12 and 24 h. The untreated cells served as control and were sampled at 0 h. The cells were collected and dissolved in Trizol and used for gene expression analysis. qPCR and statistical analysis were conducted as described above.

### Functional validation of *AjNOS*, *Ajarginase* and *Ajagmatinase* in primary coelomocytes, by siRNA

Small interfering RNAs (siRNA) targeting *AjNOS*, *Ajarginase* and *Ajagmatinase* were designed and synthesized by GenePharma (Shanghai, China). Another siRNA (negative control, *NC*) that did not target any of the genes in sea cucumber transcriptome data served as the control. Two specific siRNAs were designed for each gene for RNA interference. The detailed sequences are shown in Table 1. These siRNAs were then dissolved into RNase-free water to obtain a working solution of 20 μM. For RNA interference, 2 μL siRNAs (80 nM) of *AjNOS*, *Ajarginase* and *Ajagmatinase* (Table 1, Genepharma, China) were mixed with 1 μL siRNA-Mate (GenePharma), and then transfected into 500 μL primary cultured cells in each well. The primary cells with non-targeted double-strand siRNA (Table 1) served as a control group. At 24 h post-transfection, half of the cells were harvested, dissolved in Trizol, and used for a gene silencing efficiency assay. The rest of the cells were collected for NO production and arginase activity analysis.

### Functional validation of *AjNOS*, *Ajarginase* and *Ajagmatinase in vivo* by siRNA

Sea cucumbers *A. japonicus* (weight: 82 ± 10 g) were used for *in vivo* RNAi experiments. Briefly, the six identical siRNA sequences of *AjNOS*, *Ajarginase*, and *Ajagmatinase* (Table 1) with extra 2’ Ome modification were designed for the *in vivo* assay and synthesized by GenePharma (Shanghai, China). Each siRNA and a negative control were dissolved in RNase-free water to obtain a working solution of 20 μM. We mixed 10 μL of each siRNA (400 nM) or a negative conrol with 10 μL of transfection reagent (Beyotime biotechology, China) and 80 μL of phosphate buffered solution (PBS) at pH 7.6 to serve as the transfection solution. Sea cucumbers were injected with 100 μL of transfection solution via tentacles. After 24 h injection, the treated and control coelomocytes were collected for expression analysis, NO production and arginase activity analysis. The assays described above were biologically repeated five times.

### Dose-dependent expression profiles of arginine in pathogen- or non-pathogen-challenged coelomocytes *in vitro*

*A. japonicus* primary cells were cultured in L-15 cell culture medium (Invitrogen, USA) containing penicillin (100 U mL^−1^) and streptomycin sulfate (100 mg mL^−1^) as described above. After 12 h of culturing for recovery, the culture medium was discarded, and the cells were washed two times in PBS and then replaced with antibiotic-free L-15 with live *V. splendidus*, *V. parahaemolyticus*, and *E. coli* added at appropriate multiplicites of infection (MOIs) of 10 and 100, respectively. The cultured primary cells operated as before without bacterial co-incubation served as the control. The primary cells with each bacterial co-incubation and control group were incubated at 16 °C for 12 and 24 h. Finally, the culture medium was discarded, and the cells were washed two times in PBS and collected for the detection of NO production and arginase activity.

### Measurement of NO production and arginase activity

NO production in *A. japonicus* primary cells from the stimulation and control groups were analyzed with an NO colorimetric assay kit (Jiancheng, Nanjing, China). Absorbance was measured at OD_550 nm_ using a microplate reader (Thermo Scientific). The protein concentration of cells was measured using the BCA Protein Assay Kit (Sangon, China). The NO content was expressed as μmol/gprot.

Arginase activity was measured via a colorimetric assay for the detection of urea production from L-arginine as described previously^75^,^76^ and with minor modifications. Approximately 10^5^ cells were mixed with 50 μL cell lysis buffer (Beyotime biotechology, China) and stirred for 30 min at room temperature. After the cells were lysed, the cells were centrifuged at 12,000 × g for 5 min and the supernatant was transferred to a centrifuge tube. Approximately 25 μL of 10 mM MnC1_2_ was added and the mixture was activated for 10 min at 56 °C. The mixture was incubated with 50 μL L-arginine (0.5M, pH 9.7) for one hour at 37 °C to hydrolyze the L-arginine. The hydrolysis reaction was stopped with an acid mixture containing H_2_SO4, H_3_PO4 and H_2_O (1:3:7) and the mixture was then heated at 100 °C with 25 μL of α-isonitrosopropiophenone (9% α-ISPF in absolute ethyl alcohol) for 45 min. The samples were kept in the dark at room temperature for 10 min, and the absorbance was measured at 540 nm in a microplate reader. One-way ANOVA was applied to discern significant differences between control and experimental groups. Any significant differences relative to the control for each time point were indicated by an asterisk at *P* < 0.05 and two asterisks at *P* < 0.01.

## Additional Information

**How to cite this article**: Yina, S. *et al*. The first description of complete invertebrate arginine metabolism pathways implies dose-dependent pathogen regulation in *Apostichopus japonicus*. *Sci. Rep*. **6**, 23783; doi: 10.1038/srep23783 (2016).

## Supplementary Material

Supplementary Information

## Figures and Tables

**Table 1:** Primers used in this study.

Primer Name	Primer Sequence (5′–3′)	Used for
*AjNOS* 3-1	CAGGTTTTTGATGCACGAAATGC	3′ RACE
*AjNOS* 3-2	CGCCAATCTCTGGAAGCATCACT	
*AjNOS* 3-3	CGAGACATTCTGCGTGGGTAAGG	
*AjNOS* 3-4	CTGACGCTGCTCGGAAAAGGTAG	
*AjNOS* 3-5	AGCGACCAAGGCAAAATGAGTAT	
*AjNOS* 5-1	GCACCAAAGATGAGTTCCATTTC	5′ RACE
*AjNOS* 5-2	ACTCGCTCTCCTTTCCTTCTACC	
*AjNOS* 5-3	CCGTAATTCCATGTGTCTCGCTCT	
*AjNOS* 5-4	TCGGCTAATGTGGTCCTCTGGTT	
*AjNOS* 5-5	GCCGTTTGAGGAGCCTTATTGAG	
*Agarginase* 3-1	ACAAGCACGGATTACCAGAAGTC	3′ RACE
*Agarginase* 3-2	ACACCAAGCACAGGCACAAGAGT	
*Agarginase* 5-1	GATTAGGTCTCCTTCCCTTATGG	5′ RACE
*Agarginase* 5-2	TGGACTGAAAAATGGGTGACAAC	
*Ajagmatinase* 3-1	ATGGAATCATCAAAGGGTCTGGA	3′ RACE
*Ajagmatinase* 3-2	TTGGTCCGAGGCAAATAAGAACT	
*Ajagmatinase* 5-1	AATGTCGGCAACTCGTAAGGATG	5′ RACE
*Ajagmatinase* 5-2	ATTCCAACAAAGCAGGCATCCAG	
*AjNOS* F	GTAGAAGGAAAGGAGAGCGAGTC	Real-time PCR
*AjNOS* R	CATCGTGTCTCGTCGCATAGTGT	
*Ajarginase* F	AAGCGTTGGGATTCTCGGTGTG	Real-time PCR
*Ajarginase* R	TGGGAGTTCTTCACGAGAGGTTG	
*Ajagmatinase* F	CCTTACGAGTTGCCGACATTGGT	Real-time PCR
*Ajagmatinase* R	CTCGTCAAATGCCCTACGGAATG	
*Ajβ-actin* F	CCATTCAACCCTAAAGCCAACA	Real-time PCR
*Ajβ-actin* R	ACACACCGTCTCCTGAGTCCAT	
	siRNA interference sequences	*AjNOS* interference
Sense 1	GCGACGAGACACGAUGUUUTT	
Anti-sense 1	AAACAUCGUGUCUCGUCGCTT	
Sense 2	GCUGCAGUUCACCGGUAUUTT	
Anti-sense 2	AAUACCGGUGAACUGCAGCTT	
Sense 1	GGACGAUUGUGCCGAUCAATT	*Ajarginase* interference
Anti-sense 1	UUGAUCGGCACAAUCGUCCTT	
Sense 2	GCGAGAGUACAUGCCACAATT	
Anti-sense 2	UUGUGGCAUGUACUCUCGCTT	
Sense 1	GCGGAUUCAUCGGACGAAATT	*Ajagmatinase* interference
Anti-sense 1	UUUCGUCCGAUGAAUCCGCTT	
Sense 2	CCAUCAGUGGAUUAGAUAUTT	
Anti-sense 2	AUAUCUAAUCCACUGAUGGTT	
Sense	UUCUCCGAACGUGUCACGUTT	Negative control (NC) interference
Anti-sense	ACGUGACACGUUCGGAGAATT	
