# Supplementary material for: The first description of complete invertebrate arginine metabolism pathways implies dose-dependent pathogen regulation in Apostichopus japonicus
Source: Sci Rep. 2016 Apr 1;6:23783. doi: 10.1038/srep23783 (PMC4817134; doi:10.1038/srep23783)
Supplement: Supplementary Information [file srep23783-s1.pdf]

The first description of complete invertebrate arginine metabolism pathways implies dose-dependent pathogen regulation in *Apostichopusjaponicus*

Shao Yina, Li Chenghua[†], Zhang Weiwei, Wang Zhenhui, Lv Zhimeng

School of Marine Sciences, Ningbo University

[†]Address correspondence to Chenghua Li, lichenghua@nbu.edu.cn

Supplementary Figures 1 to 7

```
   1 CTTGCGATATTTTGACCTAAAACACCTTTTTCGTTCCCCCCATTTAAGAACCCCGATCAT
   1          M  S  D  T  N  S  S  A  I  M  M  E  N  E  P  N  T  F
  61 GAAACAATATGTCTGACACAAACTCATCAGCGATAATGATGGAGAATGAACCGAATACAT
  19   S  I  R  L  L  K  R  P  P  H  G  L  G  F  I  V  R  K  R  K
 121 TCTCAATAAGGCTCCTCAAACGGCCGCCTCATGGATTGGGCTTCATAGTGCGCAAGCGCA
  39   T  N  P  P  V  V  I  S  D  L  I  P  G  G  V  A  E  Q  S  C
 181 AAACAAACCCACCGGTGGTCATCTCAGACCTCATACCAGGGGGTGTGGCTGAACAAAGCT
  59   L  V  Q  V  G  D  V  L  L  A  V  N  G  V  Q  L  T  D  V  P
 241 GCCTAGTTCAAGTTGGAGATGTACTGTTAGCTGTTAATGGTGTCCAATTAACCGATGTAC
  79   Y  N  T  A  L  E  V  L  R  S  V  P  M  D  S  P  C  V  L  V
 301 CCTACAACACCGCCTTGGAAGTCCTACGTAGTGTACCAATGGACTCACCTTGCGTTCTCG
  99   L  R  G  P  D  G  F  K  T  K  L  E  T  L  M  S  P  D  G  L
 361 TGCTACGAGGACCCGACGGGTTTAAGACCAAGTTAGAGACATTAATGTCCCCCGACGGTT
 119   V  Q  V  I  R  L  T  E  P  E  D  H  I  S  R  D  R  N  Q  N
 421 TGGTTCAGGTCATTCGCCTAACAGAACCAGAGGACCACATTAGCCGAGATCGAAACCAAA
 139   L  T  P  K  K  Q  P  R  K  N  G  P  R  S  P  G  H  T  A  N
 481 ATTTGACTCCGAAAAAGCAACCCCGCAAAAATGGCCCACGTTCCCCGGGACACACAGCGA
 159   M  S  W  P  R  N  T  H  A  N  V  D  S  I  A  N  V  Y  N  Q
 541 ACATGTCCTGGCCGCGCAACACTCATGCCAACGTGGACAGTATCGCGAACGTTTACAACC
 179   S  H  T  H  N  G  S  V  H  Y  G  V  P  A  S  N  R  G  R  S
 601 AATCCCACACCCACAACGGTTCAGTTCATTACGGTGTCCCAGCCAGCAACAGGGGAAGGA
 199   T  P  E  G  S  P  Y  R  Y  G  R  R  S  R  R  D  T  T  R  S
 661 GTACGCCGGAGGGGAGTCCCTACAGGTACGGGAGGAGATCCCGCAGGGACACCACCAGGT
 219   P  A  R  N  I  N  S  M  P  T  S  P  M  R  S  Y  N  P  I  R
 721 CACCCGCGCGCAACATTAACAGCATGCCGACAAGCCCTATGCGCAGCTACAATCCTATCC
 239   D  S  S  P  S  T  C  I  C  R  G  H  F  M  N  T  E  P  Q  Q
 781 GCGATTCCTCCCCATCGACTTGTATCTGTCGAGGTCATTTCATGAATACGGAACCTCAAC
 259   V  L  A  W  N  G  T  F  P  R  D  V  Y  N  M  R  I  S  P  E
 841 AAGTTCTGGCCTGGAATGGAACTTTCCCCCGTGACGTGTACAACATGAGGATATCCCCGG
 279   E  I  Y  F  D  S  P  R  T  S  I  D  Y  D  L  E  N  E  R  I
 901 AAGAGATTTACTTCGACAGCCCCCGCACCTCGATTGACTATGACCTTGAGAATGAACGCA
 299   V  M  N  G  V  A  N  H  T  P  S  V  I  G  E  P  S  E  L  V
 961 TCGTTATGAATGGGGTGGCAAATCACACCCCCTCTGTCATCGGTGAACCCTCCGAGCTAG
 319   T  E  S  I  G  T  Q  W  P  E  P  I  I  T  A  K  N  K  G  E
1021 TAACCGAATCGATTGGTACACAATGGCCTGAGCCAATCATCACAGCAAAGAATAAAGGGG
 339   G  S  S  D  E  W  A  K  S  E  N  G  I  T  I  S  G  K  A  N
1081 AGGGCTCAAGCGACGAATGGGCAAAGTCCGAGAACGGAATCACCATATCGGGCAAAGCCA
 359   T  G  V  A  N  S  G  I  S  Q  V  D  D  V  G  G  T  H  E  Q
1141 ACACCGGTGTGGCCAACAGCGGCATTTCACAAGTGGATGACGTAGGCGGTACTCATGAAC
 379   H  V  V  D  A  E  D  K  Q  R  K  V  A  T  R  G  D  Q  T  G
1201 AACACGTCGTTGATGCCGAGGACAAACAGAGAAAGGTCGCGACGAGAGGGGATCAAACAG
 399   T  T  I  T  T  S  G  I  K  E  Q  E  I  A  N  S  V  Q  S  S
1261 GTACTACTATTACTACCAGTGGGATTAAAGAGCAGGAGATCGCAAATTCAGTGCAAAGTA
 419   R  L  P  E  G  T  M  G  A  G  C  S  T  V  P  K  T  G  V  V
```

1321 GTAGGCTGCCGGAGGGTACAATGGGAGCAGGTTGTTCGACCGTACCCAAAACCGGAGTTG
439 S I T P S S T E T S T A T I N Q V N L Q
1381 TTTCAATAACACCGTCGAGCACGGAGACTAGCACTGCGACGATTAATCAAGTTAATTTAC
459 H V G N D K Q N G I T V A Q S E T H G I
1441 AGCACGTTGGTAATGATAAACAAAATGGCATTACTGTTGCCCAGAGCGAGACACATGGAA
479 T D T P E K K P T I A R Q N A V N R Q Q
1501 TTACGGATACCCCGGAGAAGAAACCGACGATTGCGCGTCAAAACGCCGTGAATCGGCAAC
499 T K E T T T R N T T T T T T T G E M I N
1561 AGACGAAGGAGACAACAACTCGTAATACTACTACCACTACTACTACTGGGGAAATGATAA
519 G H S N G T T D S H D M V L E E Q V V K
1621 ACGGACATAGCAACGGTACTACAGATAGTCATGATATGGTACTGGAGGAGCAAGTCGTCA
539 Q N G G L P I T A D N N M R E V K L N D
1681 AACAGAATGGGGGGCTGCCGATAACTGCCGATAACAACATGCGAGAGGTAAAACTCAACG
559 A G V Q T E T S K V K Q N G E E V E G K
1741 ACGCAGGGGTGCAGACGGAGACCTCTAAAGTTAAGCAGAACGGAGAAGAGGTAGAAGGAA
579 E S E S D T Q G T H G A V C A R E K S K
1801 AGGAGAGCGAGTCGGACACACAGGGTACGCATGGGGCCGTGTGTGCCCGGGAGAAATCCA
599 Q K Y V R V T N L I D G K Q F T D T L H
1861 AACAGAAGTACGTCAGGGTCACGAATCTGATAGATGGGAAACAGTTTACGGATACACTTC
619 Q K T Y I K T L C D E T R C F G S I M F
1921 ATCAAAAGACTTACATCAAAACACTATGCGACGAGACACGATGTTTCGGATCGATTATGT
639 P P K G K G R S K D E V L L Q A K D F I
1981 TCCCGCCAAAAGGCAAAGGACGAAGCAAGGACGAGGTTCTACTCCAAGCAAAGGATTTCA
659 R Q Y F T H I K R A D T P A H K K R W D
2041 TCCGCCAGTACTTCACTCATATAAAAAGAGCTGACACACCCGCACACAAGAAAAGGTGGG
679 E V E S S I E Q K G I Y D L T E M E L I
2101 ATGAAGTTGAAAGTAGCATTGAACAAAAGGGTATCTATGACCTCACTGAAATGGAACTCA
699 F G A K T A W R N A P R C I G R I Q W T
2161 TCTTTGGTGCTAAAACAGCATGGAGGAACGCACCGCGGTGTATCGGCAGAATCCAGTGGA
719 K L Q V F D A R N A T T A H D M Y H A I
2221 CCAAACTACAGGTTTTTGATGCACGAAATGCGACAACCGCTCATGATATGTACCACGCAA
739 C N H M G Y A T N K G N L R S A I T I F
2281 TATGTAACCACATGGGCTATGCAACAAATAAGGGCAACTTGAGATCGGCGATCACAATTT
759 P P R T D G E H D F R V W N P Q L I R Y
2341 TCCCGCCGAGAACAGACGGTGAACACGATTTTCGAGTATGGAATCCACAGCTGATACGAT
779 A C Y K Q P D G K L I G D P A N L E F T
2401 ACGCTTGCTACAAGCAACCAGATGGCAAACTTATAGGTGATCCCGCTAATCTCGAGTTCA
799 E V C Q K L G W K G S G G A W D I L P L
2461 CGGAGGTTTGCCAAAAACTTGGATGGAAGGGGAGTGGGGGAGCTTGGGATATATTACCTC
819 V L Q A N G Q D P E V Y D I P Q E L I L
2521 TCGTTCTACAAGCTAACGGCCAAGATCCAGAGGTCTATGATATACCTCAAGAATTGATCC
839 E V H M S H P N Y D W F A D L G L R W Y
2581 TGGAGGTGCACATGTCACATCCAAATTATGACTGGTTTGCCGACCTAGGTTTGAGGTGGT
859 A L P A V S C I M L D L G G L Q F T G I

2641 ACGCTCTTCCAGCAGTCTCTTGCATCATGCTCGATCTCGGAGGGCTGCAGTTCACCGGTA

879 P F N G W Y M E T E I G A R D F C D E N

2701 TTCCGTTCAACGGATGGTACATGGAGACTGAAATAGGAGCTAGAGACTTCTGCGATGAAA

899 R L N M C E T I A R K M G L D V S T N A

2761 ACAGGCTGAATATGTGCGAGACCATTGCAAGGAAGATGGGCCTTGATGTCAGCACGAACG

919 T L W K D K A V V E A N V A V M H S F Q

2821 CAACCCTGTGGAAGGATAAGGCAGTAGTAGAAGCCAACGTAGCGGTCATGCACAGCTTTC

939 K A N V T I T D H H S A S E S F L K H M

2881 AGAAAGCAAATGTCACAATCACGGATCATCACTCCGCATCGGAATCATTCCTGAAACACA

959 E N E Q R L R G G C P A D W V W I V P P

2941 TGGAGAACGAGCAGAGGTTGCGAGGAGGCTGTCCTGCCGATTGGGTGTGGATCGTACCGC

979 I S G S I T P V F H Q E M L S Y N L K P

3001 CAATCTCTGGAAGCATCACTCCTGTATTTCACCAAGAAATGCTCTCGTACAACCTCAAAC

999 S Y E Y Q E E P W R T H V F K T A E E G

3061 CATCCTACGAATACCAGGAAGAACCATGGAGGACTCACGTGTTCAAAACTGCCGAGGAGG

1019 Q K K K R R K L L T F K E V A K A V K F

3121 GTCAAAAGAAGAAGAGGAGGAAACTACTGACTTTCAAAGAAGTTGCAAAGGCCGTAAAGT

1039 S A K L M G K A L A K R I K A T I L Y A

3181 TCTCAGCAAAGTTAATGGGAAAAGCTTTGGCCAAACGAATCAAGGCTACCATCCTGTATG

1059 S E T G R S E K Y A K V L C E I F K H A

3241 CCTCAGAGACTGGAAGATCGGAGAAATACGCCAAGGTGCTCTGCGAGATATTCAAGCATG

1079 F D A K V L C M E D Y D P V H L E H E T

3301 CATTCGATGCCAAGGTCTTGTGCATGGAAGACTACGACCCAGTCCACCTGGAACACGAGA

1099 L L L V V T S T F G N G D P P E N G E E

3361 CGCTCCTGCTAGTCGTTACGAGTACCTTTGGAAACGGTGATCCGCCAGAGAATGGAGAGG

1119 F G R F L M E L R R D H T G S E D S S K

3421 AGTTTGGTCGGTTTCTGATGGAACTTCGAAGGGACCACACAGGGTCCGAGGACAGCTCCA

1139 A L R F D K F A S E D R E E K A V S K R

3481 AAGCTCTACGGTTTGACAAGTTTGCTTCTGAGGATAGAGAAGAGAAGGCCGTATCCAAGC

1159 A S A N L D S S S G P L A N V R Y S I F

3541 GAGCCTCGGCAAACCTCGATTCCAGTTCCGGGCCATTGGCTAATGTGAGGTACTCCATCT

1179 G L G S S A Y P N F C A F A K A V D P L

3601 TTGGCCTCGGATCCAGCGCGTATCCTAACTTTTGCGCGTTCGCCAAAGCCGTGGATCCTC

1199 I A D M D G E R I Q A L G E G D E L S G

3661 TGATTGCGGACATGGACGGCGAGAGGATTCAGGCCCTCGGCGAGGGAGACGAACTCAGTG

1219 Q E E S F R T W A K A V F K A A C E T F

3721 GCCAGGAAGAGTCCTTCAGGACCTGGGCCAAGGCTGTATTTAAGGCCGCCTGCGAGACAT

1239 C V G K D V N M Q E A N A S L Q T N E L

3781 TCTGCGTGGGTAAGGATGTGAACATGCAAGAGGCGAACGCCTCCCTGCAGACCAACGAAC

1259 S W A P D K F R L V P E E S G T A M E L

3841 TCAGCTGGGCTCCGGACAAGTTCAGGTTGGTCCCGGAAGAGAGCGGAACCGCCATGGAAT

1279 C E A L T K L H T K R V L P C L L M S S

3901 TATGCGAAGCCCTCACTAAGCTGCACACCAAGAGAGTGTTGCCGTGTCTGCTTATGTCGA

1299 N D L Q S D E S T R S T I Q V T M D T Q

3961 GCAACGATCTCCAATCAGACGAATCCACTCGCTCAACTATACAAGTCACGATGGATACCC
1319 G A S E L L F E P G D H V A I C P A N E
4021 AGGGTGCTTCAGAACTGCTATTCGAACCCGGTGATCATGTCGCCATCTGCCCTGCCAACG
1339 K A L V D A I L A R V K N E R T P D E V
4081 AGAAGGCCCTGGTGGATGCCATCCTGGCCAGAGTCAAGAACGAACGGACACCGGATGAAG
1359 V V V Q V L Q Q R S T P L G V V K A W N
4141 TGGTGGTAGTACAGGTGCTTCAGCAGAGATCGACACCGTTAGGTGTGGTCAAAGCCTGGA
1379 P T E R L P P C S M R Q A L E Y Y V D I
4201 ACCCCACAGAGAGGCTTCCTCCTTGTAGCATGAGGCAAGCCTTGGAGTATTACGTGGACA
1399 T T P P S P D F L R L L S L Q A T D P K
4261 TCACCACGCCCCCTTCACCGGACTTCCTGCGCCTACTCAGTTTACAGGCCACAGACCCGA
1419 E Q E K L T L L G K G S S A Y E D W K Y
4321 AGGAACAAGAAAAGCTGACGCTGCTCGGAAAAGGTAGCAGTGCTTATGAAGACTGGAAGT
1439 E N S P N L I E V L N E F S S I N L S I
4381 ACGAGAACAGCCCGAATCTGATAGAGGTCTTGAACGAGTTTTCCTCGATCAATCTGTCGA
1459 E F L L Q E A L L L K P R Y Y S I S S S
4441 TCGAGTTTCTGCTGCAGGAGGCTCTCCTGCTCAAGCCACGTTACTACTCCATCAGCTCCA
1479 L R M Y P G E I H A T V A V V C W R T K
4501 GCCTGAGGATGTACCCCGGAGAGATACATGCCACCGTGGCTGTTGTCTGTTGGCGGACTA
1499 G G K G P L H N G V C S S W L N R L K P
4561 AAGGTGGTAAAGGCCCCCTACACAACGGTGTCTGCTCTTCCTGGTTGAATCGACTGAAAC
1519 G E V I P A F I R Q A P L F R L P E K R
4621 CAGGAGAAGTTATCCCAGCTTTCATCAGACAAGCACCCCTGTTCCGCCTCCCAGAGAAGA
1539 N L P C I L V G P G T G I A P F R S F W
4681 GGAACCTGCCGTGCATCCTGGTGGGTCCCGGAACGGGAATCGCTCCCTTCAGATCCTTCT
1559 Q Q R Q I D L Q F T S A V N E K G E K V
4741 GGCAGCAACGGCAGATCGACCTGCAGTTCACCAGCGCCGTCAACGAGAAGGGAGAGAAGG
1579 L G D I S M F F G C R H P N L D N I Y A
4801 TCCTGGGAGACATCTCCATGTTCTTCGGGTGCAGGCATCCTAACTTAGATAACATCTACG
1599 E E K Q K A I T D G A L K E V H I A Y S
4861 CCGAGGAGAAACAGAAGGCCATCACAGACGGCGCTCTGAAGGAAGTGCACATTGCGTACT
1619 R E P E Q P K K Y V Q H V L K D Q A R E
4921 CCAGAGAGCCCGAACAGCCAAAGAAATATGTCCAACACGTCCTGAAAGACCAGGCTCGTG
1639 V F R T L D Q D N G H F F V C G D V S M
4981 AAGTCTTCAGGACTCTAGATCAGGACAATGGACATTTCTTCGTCTGCGGGGACGTCTCCA
1659 A G D V Q K A L E E I I S D Q G K M S I
5041 TGGCGGGGGACGTCCAGAAGGCCCTTGAGGAGATCATCAGCGACCAAGGCAAAATGAGTA
1679 D Q A K E Y V S K L R D H T R Y H E D I
5101 TAGATCAAGCTAAAGAATACGTCAGCAAATTGAGGGACCACACCCGTTACCACGAGGACA
1699 F G V T L K T A E V R S K L R T A V R A
5161 TCTTTGGGGTCACGCTGAAGACAGCAGAGGTGAGGTCCAAGCTCCGCACGGCCGTCCGGG
1719 H V L I S A G G S K L T P A G E A L Q A
5221 CCCACGTATTGATCAGCGCTGGTGGCAGCAAACTCACCCCTGCGGGAGAGGCCCTCCAGG
1739 D Q N E N G Q D G P N K E L E R S N S V

5281 CCGATCAGAACGAAAATGGCCAAGATGGACCAAACAAGGAACTGGAAAGATCCAACTCTG
1759 K S L G D K E K R E S P *
5341 TGAAATCACTTGGAGATAAAGAGAAAAGAGAAAGCCCA**TAA**ATTGGAGATGAAGAAAAAG
5401 GAACCAATCAAAACACCCAACGGCGACATTCCCAAGGTGAACGTGAAAAAGGTTTAGTAC
5461 GTAGAAACAAACTGGATGCTTTTGTCGATGGGATGGTCTTTGAGATCTGTTTGAGATCTA
5521 CGGTTCATCATCTTCATCTTCTCCAGCCGGTCTCCTCTGATAGATTGAGAATATAGACCA
5581 ACACTATAGTGACCTCTGACCTGTCGGGGTCGATGAATTAACGAAGCGAGGGGCGAAAGG
5641 AATTAGCTGAGAGGAATGACTGGGTGTGTGTGTCTTGGGAAGCTGTAAAAAATTTGCAAC
5701 ATGAAGAAGATGTTTGTTTTTAAATATTGCTTCGTAATCTCCACTTTTTCACAGATTTTA
5761 AATGTTTGAAACCTCTGAGCACAAAAAAAAAGTATAAGGAAAAACATGACATGAAAAGGC
5821 CAAAAAACCC***ATTTA***GACATGG***AATAAA***AAAAAGAAGGAATTGTCGACAAGTTGCAGATT
5881 TTACTGTACAGAAACTCATGGCAACATTATATTATCCGTTACAAATTTACATATTTGTGA
5941 ATATTAAAAAAAAAAAA

Supplementary Figure 1 |The complete nucleotide and deduced amino acid sequence of *Apostichopus japonicus*NOS. The start codon was blacked. The asterisk indicated the stop codon. Predicted PDZ domain was indicated by gray shadow, an NO synthase domain was double underlined, a flavodoxin 1 domain sequence was bold underlined and a FAD binding domain sequence was underlined, an NAD-binding domain was indicated in wavy line. The RNA instability sequences (ATTTA) and polyadenylation signal (AATAAA) were indicated by bold italics.

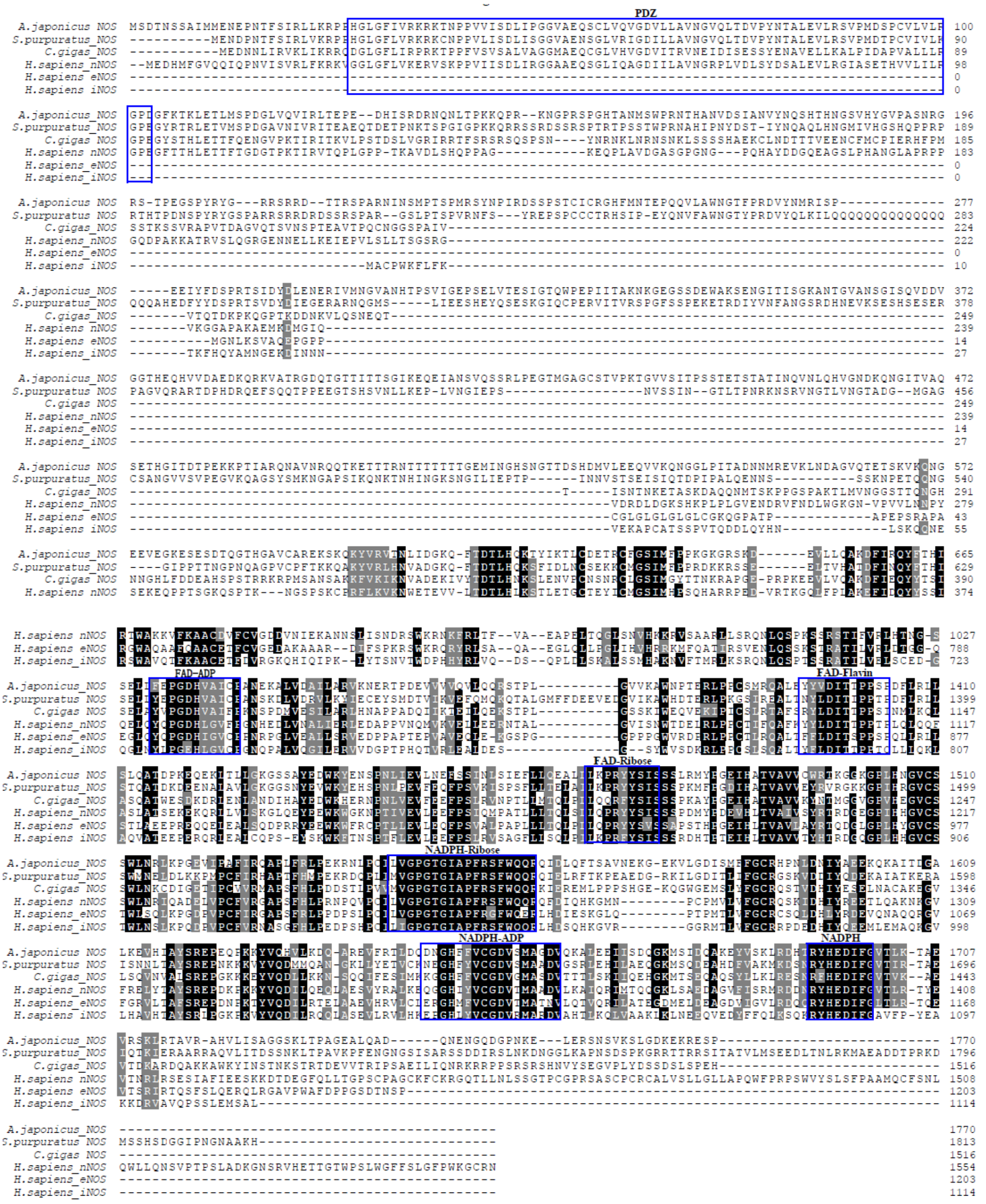

Supplementary Figure 2 |Alignment of the predicted amino acid sequences of NOSs using ClustalW2 Multiple Alignment program. The consensus residues were shaded with a threshold of more than 80% identity using Multiple Align Show program. Identical residues were indicated in black, and similar residues in light gray. Domains of NOS proteins were predicted by SMART program and highlighted by solid-lined boxes, including PDZ domain, NO synthase domain (contain Heme and BH_4), Flavodoxin 1 domain (contain CaM, FMN-1, FMN-2, Autoinhibitory loop and FMN-3), FAD binding domain (contain FAD-ADP, FAD-Flavin and FAD-Ribose) and NAD binding domain (contain NADPH-Ribose, NADPH-ADP and NADPH). Cysteine residue in NO synthase catalyzed reactions domain was indicated directly

below the alignments. The accession numbers of these proteins from GenBank were as follows: *Strongylocentrotus purpuratus* NOS (XP_011665134.1), *Crassostrea gigas* NOS (XP_011420158.1), *Homo sapiens* nNOS (AAB60654.1), *Homo sapiens* eNOS (AAH69465.1), *Homo sapiens* iNOS (BAA37123.1).

1 CGCTTCTGATCCTGGTGACTATCCATATGCTAATCATCTTGTTATTTATGTGACATGTTG

61 ACACAGATCCAAGTGTACTAACGGTACTGTCCGTAGAGTTGAAAGGGAACAGAACTGAAT

121 GAAAACCGGTGTGTTCCAACCACAAGGAGCAGAAGCTATATACGTACATATATTCGGTTT

181 GGGATTCTGTAGCCTCAAGTTTCGGGGTTGATCGTGAAAACGTGCTTAAAATAGCAAAAA

241 CTTGCAGGACTTGCAGGTTTTTCTGCTGCAGGCCGAGAAACAAATTGCTCACGTACTGTT

1 M F K M A A L S M K R M V S K G C K S

301 CAC**ATG**TTTAAAATGGCTGCTTTATCCATGAAACGGATGGTTTCAAAGGGGTGTAAATCC

20 S V S S L L S P I F Q S S K G V K H A P

361 TCAGTTTCATCGTTGTTGTCACCCATTTTTCAGTCCAGCAAAGGGGTGAAACATGCCCCT

40 H S F G K D F S T V N K T G K R S V G I

421 CATTCTTTTGGGAAAGATTTCAGCACAGTGAATAAAACAGGCAAGAGAAGCGTTGGGATT

60 L G V P I N R G Q T N P G V L Y G P A A

481 CTCGGTGTGCCTATTAACAGAGGACAGACTAATCCTGGGGTGTTGTATGGCCCTGCTGCC

80 I R E G D L I K R I K G Q W H E I H D H

541 ATAAGGGAAGGAGACCTAATCAAAAGAATCAAAGGACAATGGCATGAAATTCACGATCAC

100 G D L G F D Y V P E D D C A D Q P L V K

601 GGTGATCTCGGATTCGACTATGTGCCGGAGGACGATTGTGCCGATCAACCTCTCGTGAAG

120 N S H S L G R A L Q K V S S S V Q T I V

661 AACTCCCACTCACTAGGTAGAGCATTGCAAAAGGTTTCTTCATCTGTCCAGACCATTGTA

140 S A G E V C I V V G G D H S L S T G S I

721 TCAGCAGGTGAGGTTTGCATCGTTGTGGGTGGGGATCATAGCCTATCGACAGGATCCATT

160 H G H S L A E P D I C L L W I D A H A D

781 CATGGTCACAGCCTGGCTGAACCAGATATTTGTTTGTTGTGGATCGACGCCCACGCCGAC

180 I N L P M T S P S G N M H G M V L S F C

841 ATCAATCTACCCATGACATCTCCGTCGGGAAATATGCACGGCATGGTCCTGTCCTTTTGC

200 V K E L R E Y M P Q I P G L E W L K P T

901 GTCAAAGAACTGCGAGAGTACATGCCACAAATCCCTGGCCTAGAATGGTTGAAGCCGACA

220 I S A K N I A F I G L R D L D A G E K Y

961 ATATCTGCAAAGAATATCGCTTTTATTGGCCTGAGGGACCTAGATGCAGGAGAAAAATAC

240 I I D Q L N M T A Y S I H D V D K H G L

1021 ATCATAGATCAGCTCAACATGACAGCATATTCCATCCATGATGTTGACAAGCACGGATTA

260 P E V L A R S M D A I N P R G N R P I H

1081 CCAGAAGTCCTGGCCAGGTCTATGGATGCCATCAATCCAAGGGGAAATCGACCAATTCAT

280 L S F D V D S L D P S V T P S T G T R V

1141 CTCAGTTTTGACGTGGACAGTTTGGATCCGTCCGTTACACCAAGCACAGGCACAAGAGTT

300 A G G L T F R E G M Y I V E E V A N T G

1201 GCTGGTGGGCTCACCTTTAGAGAAGGAATGTACATTGTAGAAGAAGTGGCAAATACTGGA

320 M M S A M D L V E V N P T I G T Q A D K

1261 ATGATGAGTGCCATGGATTTGGTGGAGGTGAACCCAACCATTGGTACCCAAGCCGACAAA

340 E R T I D C A I R L V G G A L G K K R E

1321 GAACGTACCATCGACTGTGCCATCAGGCTAGTTGGCGGTGCTCTTGGCAAGAAGCGAGAG

360 G E Y P Q N Y Q L P T V E E F L K D K K

1381 GGTGAATATCCACAAAATTATCAGCTGCCTACTGTGGAAGAATTTCTGAAAGACAAGAAA

380 P A I L K *

1441 CCTGCCATTTTGAAA**TAG**AAGCAGAGAAATGAAGCAATGACTTTGTTGAAATATTGATCT
1501 ACTGTAGCTCAGCTGTCGAAGATAATTTTTCCAAAAGTCACAAAGGTATAACTATATCTA
1561 TGTTTTCTTGGGAACGAGGCAGTATGTAAGCTATATTCGTCGAACTACAAAGTATACACT
1621 GCTGTTTTAAAGCATGCCAAAGTTGCAAACCTACAAATATATGTAGCCTGTCAGCAATCT
1681 TTAGTATGAAGCAGTGC***ATTTA***TTATTTTTATTCATAATATTTGCCATACTTGGATATAT
1741 ATTAACTGCAGAAGAAGTTATCAAAACATTGTATGTTCTTTCATTTTGCCTTTAACCACT
1801 ACCCACCTTCATAGAAACAAAACAAATATTTCAAACTTAAAGGACAAGAAAAAAAAAAAA

Supplementary Figure 3 |The complete nucleotide and deduced amino acid sequence of *Apostichopus japonicus*arginase. The start codon was blacked. The asterisk indicated the stop codon. Predicted arginase domain was indicated by gray shadow. The RNA instability sequences (ATTTA) were indicated by bold italics.

1 GAAAATGTATACTTGTTTACGGGCAGCGTGCATTTTTGAGCTAAAGTAAAGTCTTAAATC

61 AATCACAACTTGACTCAAAGCGCAAAATTAGCAGTCATAATTTATGACTTAACCTTTCTT

121 AGCCCTCAACACAGGTTAAGTGTCTCTCGTACTCGGCAGTGAATGTTTTTGACATGTGAC

181 GAGCATTTTCTTAGAAGTTAGCCAGTTTGCAGCACTTTGACAACATCGAATTAGGATTCT

1 M H R L S N Q L L R W S R P S L L A

241 GAGAAG**ATG**CATCGGTTGTCTAATCAACTACTCAGGTGGTCTCGACCAAGTCTTCTTGCG

19 Q G I S R P I S K N I R E Q S S H D R K

301 CAGGGGATCAGTAGGCCTATCTCAAAGAATATTAGAGAGCAAAGTAGCCATGACAGGAAA

39 E F F K P I S G L D M P R A G G I A T M

361 GAATTCTTCAAACCCATCAGTGGATTAGATATGCCACGTGCTGGTGGTATAGCCACCATG

59 M R L P H M E S S K G L D A C F V G I P

421 ATGCGTCTCCCACACATGGAATCATCAAAGGGTCTGGATGCCTGCTTTGTTGGAATTCCA

79 F D S G C S N R T G T R L G P R Q I R T

481 TTCGACAGCGGATGCTCTAACAGGACAGGAACTAGACTTGGTCCGAGGCAAATAAGAACT

99 E S C L L R E Y N T Q G A A P F A S L R

541 GAATCCTGTCTACTAAGAGAGTACAACACACAAGGTGCTGCTCCTTTTGCATCCTTACGA

119 V A D I G D V S L S M Y N I K K A C E S

601 GTTGCCGACATTGGTGATGTCTCACTATCAATGTACAACATCAAGAAAGCGTGTGAGAGC

139 I R K K Y K E I L E N D C V P L T L G G

661 ATCAGAAAGAAATATAAGGAAATCTTGGAGAATGACTGCGTACCACTGACTTTGGGCGGT

159 D H T I S Y P I L Q A I K E K H G P V G

721 GATCATACCATATCATATCCCATACTACAAGCTATTAAGGAGAAGCATGGTCCTGTGGGC

179 L I H V D A H A D S S D E M L G E K I A

781 TTGATACATGTCGATGCGCATGCGGATTCATCGGACGAAATGCTCGGAGAAAAGATCGCT

199 H G T P F R R A F D E G L I I P E K T I

841 CACGGTACCCCATTCCGTAGGGCATTTGACGAGGGATTAATCATCCCGGAAAAGACGATC

219 Q I G L R G S Q Y S V K D G G Q S W Q V

901 CAGATCGGACTGAGAGGGTCTCAGTATTCCGTTAAGGATGGTGGACAATCGTGGCAAGTG

239 E Q G F R V V P A E E C W Y K S M S P L

961 GAGCAGGGGTTTCGAGTGGTTCCAGCTGAAGAGTGTTGGTATAAGTCAATGTCACCGTTG

259 M A E V R D M M G Q V P V Y L S F D I D

1021 ATGGCTGAAGTCAGAGACATGATGGGACAAGTACCAGTTTATCTCTCCTTTGATATTGAT

279 A L D P A F A P G T G T P E I G G L T S

1081 GCTTTAGATCCAGCATTTGCACCAGGAACAGGTACCCCAGAGATTGGCGGGCTGACCTCC

299 I Q G L E I I R G C K G I K L V G A D L

1141 ATCCAAGGCTTAGAGATAATCAGAGGTTGCAAAGGAATCAAGTTAGTTGGCGCAGATCTT

319 V E V S P P Y D T N G T T A L T G A N L

1201 GTTGAAGTTTCACCCCCTTATGACACCAACGGAACTACTGCCTTGACGGGAGCCAATCTT

339 L F E M L C V L P G V K Y S D *

1261 CTGTTTGAAATGCTTTGTGTCTTGCCAGGTGTAAAATACTCTGAT**TGA**GTCAGCCATATT

1321 ATCAAATCATTTTCTATTAGCACAAAGTATTCAAAATACAGTATGATT*ATTTA*TAATACA

1381 GAATGATGTAGCAAAACCTAAAAAAAAAAAA

Supplementary Figure 4 |The complete nucleotide and deduced amino acid sequence

of *Apostichopus japonicus*agmatinase. The start codon was blacked. The asterisk indicated the stop codon. Predicted arginase domain was indicated by gray shadow. The RNA instability sequences (ATTTA) were indicated by bold italics.

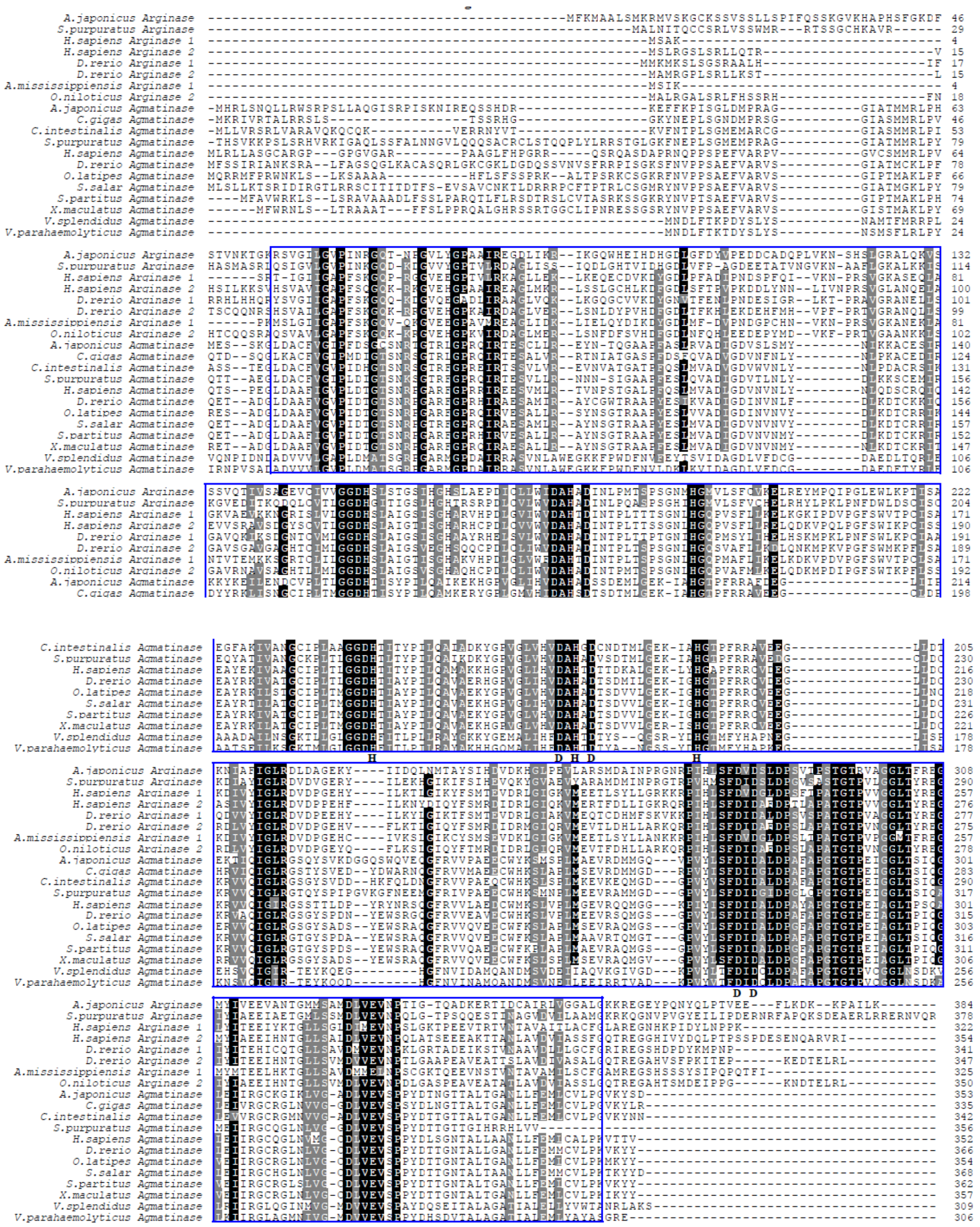

Supplementary Figure 5 |Alignment of the predicted amino acid sequences of arginases and agmatinases using ClustalW2 Multiple Alignment program. The consensus residues were shaded with a threshold of more than 80% identity using Multiple Align Show program. Identical residues were indicated in black, and similar residues in light gray. The solid-linedbox showed the arginase domain. The seven conserved Histidine (H) and Aspartic acid (D) residues required for manganese-ion-binding sites and enzymatic activity were indicated directly below the alignments. The accession numbers of these proteins from GenBank were as follows: *Crassostrea gigas*agmatinase (EKC27784.1), *Ciona intestinalis*agmatinase

(XP_002122881.1), *Strongylocentrotus purpuratus* agmatinase (XP_011669843.1), *Homo sapiens*agmatinase (AAL24446.1), *Danio rerio*agmatinase (NP_001071019.1), *Vibrio splendidus*agmatinase (WP_004733508.1), *Vibrio parahaemolyticus*agmatinase (KLI86552.1), *Stegastes partitus* agmatinase (XP_008291406.1), *Xiphophorus maculatus* agmatinase (XP_005799024.1), *Oryzias latipes* agmatinase (XP_004070948.1), *Salmo sala*r agmatinase (ACI32959.1), *Homo sapiens*arginase 1 (NP_000036.2), *Homo sapiens*arginase 2 (NP_001163.1), *Strongylocentrotus purpuratus*arginase (XP_011674364.1), *Danio rerio*arginase 1 (NP_001038662.1), *Danio rerio*arginase 2 (NP_955905.1). *Alligator mississippiensis*arginase 1 (XP_006259832.1), *Oreochromis niloticus*arginase 2 (XP_003445522.1).

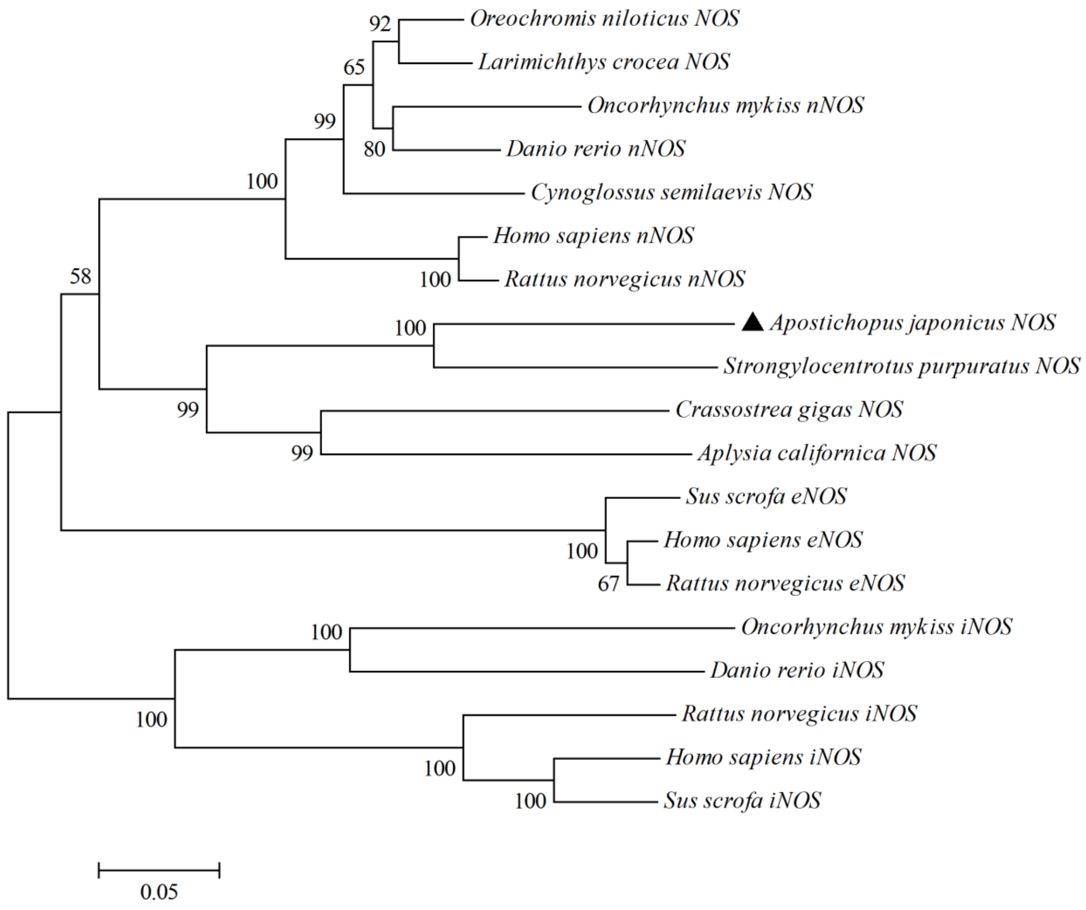

Supplementary Figure 6 |A phylogenetic tree based on the amino acid sequences of the known NOSs from other originals. The protein sequences accession numbers: *Homo sapiens* nNOS (AAB60654.1), *Homo sapiens* eNOS (AAH69465.1), *Homo sapiens* iNOS (BAA37123.1), *Rattus norvegicus* nNOS (AAC52782.1), *Rattus norvegicus* eNOS (NP_068610.1), *Rattus norvegicus* iNOS (CAB46089.1), *Sus scrofa* eNOS (NP_999460.1), *Sus scrofa* iNOS (NP_001137162.1), *Danio rerio* nNOS (AAO53340.1), *Danio rerio* iNOS (CAJ29886.1), *Oncorhynchus mykiss* nNOS (ABG24205.1), *Oncorhynchus mykiss* iNOS (CAC82808.1), *Aplysia californica* NOS (NP_001191470.1), *Cynoglossus semilaevis* NOS (XP_008334475.1), *Larimichthys crocea* NOS (XP_010735156.1), *Oreochromis niloticus* NOS (XP_003454198.2), *Crassostrea gigas* NOS (XP_011420158.1), *Strongylocentrotus purpuratus* NOS (XP_011665134.1). The tree was obtained by bootstrap analysis with Neighbor-Joining method and the numbers on the branches represent bootstrap values for 1000 replications.

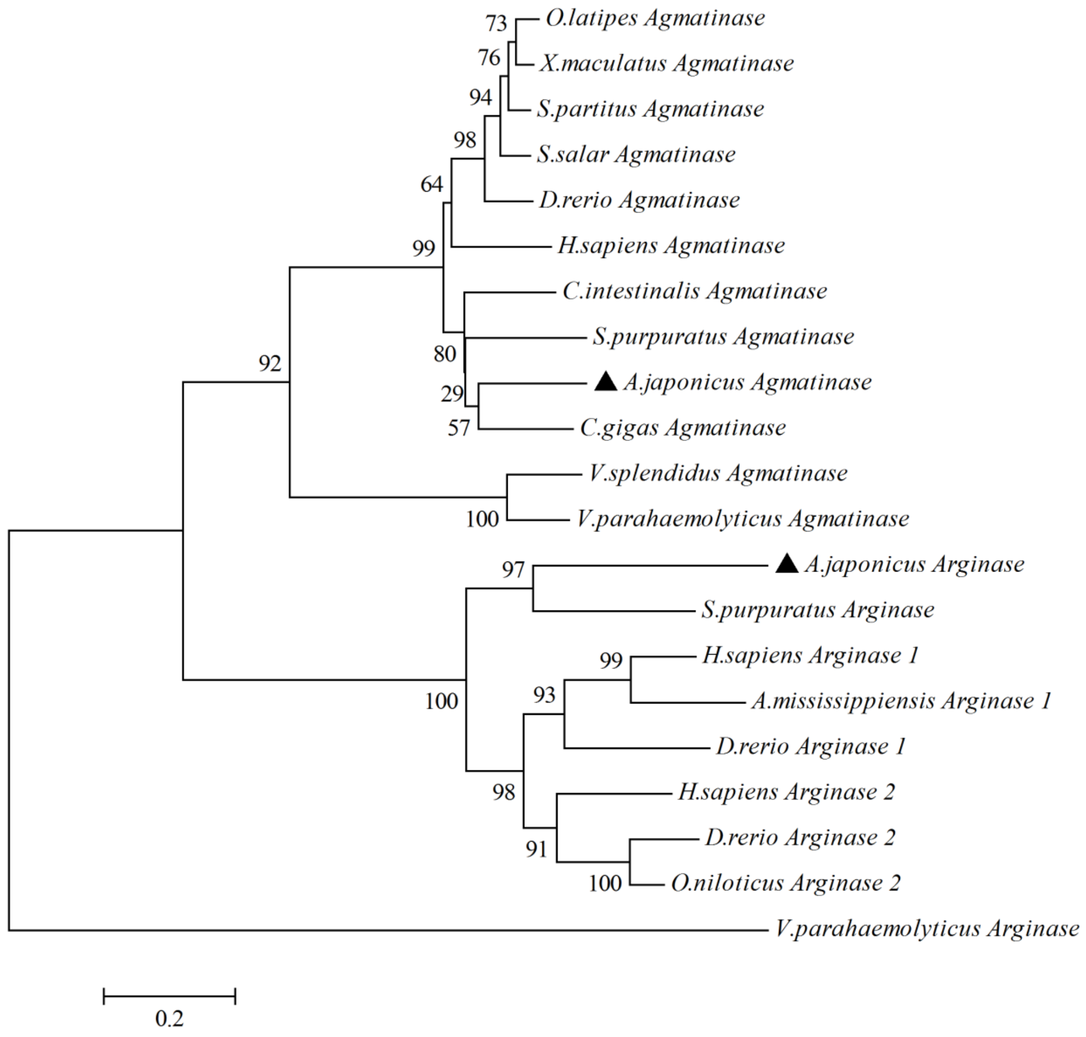

Supplementary Figure 7 |A phylogenetic tree based on the amino acid sequences of the known arginase family from other originals. The *Vibrio parahaemolyticus*arginase (WP_025791326.1) was used as out group.The protein sequences accession numbers: *Homo sapiens* agmatinase *(*AAL24446.1), *Rattus norvegicus*agmatinase (NP_001041650.1), *Danio rerio*agmatinase (NP_001071019.1), *Stegastes partitus*agmatinase (XP_008291406.1), *Xiphophorus maculatus*agmatinase (XP_005799024.1), *Oryzias latipes*agmatinase (XP_004070948.1), *Salmo salar*agmatinase (ACI32959.1), *Crassostrea gigas*agmatinase (EKC27784.1), *Ciona intestinalis*agmatinase (XP_002122881.1), *Strongylocentrotus purpuratus*agmatinase (XP_011669843.1), *Vibrio splendidus*agmatinase (WP_004733508.1), *Vibrio parahaemolyticus*agmatinase (KLI86552.1), *Homo sapiens* arginase 1 (NP_000036.2), *Homo sapiens*arginase 2 (NP_001163.1), *Danio rerio*arginase 1 (NP_001038662.1), *Danio rerio*arginase 2 (NP_955905.1), *Crassostrea gigas*arginase (XP_011443425.1), *Strongylocentrotus purpuratus*arginase (XP_011443425.1). The tree was obtained by bootstrap analysis with Neighbor-Joining method and the numbers on the branches represent bootstrap values for 1000 replications.
